# HNF4α ubiquitination mediated by Peli1 impairs FAO and accelerates pressure overload-induced myocardial hypertrophy

**DOI:** 10.1038/s41419-024-06470-7

**Published:** 2024-02-12

**Authors:** Yuxing Hou, Pengxi Shi, Haiyang Du, Chenghao Zhu, Chao Tang, Linli Que, Guoqing Zhu, Li Liu, Qi Chen, Chuanfu Li, Guoqiang Shao, Yuehua Li, Jiantao Li

**Affiliations:** 1 Key Laboratory of Targeted Intervention of Cardiovascular Disease, Collaborative Innovation Center for Cardiovascular Disease Translational Medicine, School of Basic Medical Science, Nanjing Medical University, Nanjing, 211166 China; 2 Department of Pathology and Pathophysiology, School of Medicine & Holistic Integrative Medicine, Nanjing University of Chinese Medicine, Nanjing, 210023 China; 3 Key Laboratory of Targeted Intervention of Cardiovascular Disease, Department of Physiology, Nanjing Medical University, Nanjing, 211166 China; 4 Department of Geriatrics, The First Affiliated Hospital of Nanjing Medical University, Nanjing, 210029 China; 5 Department of Surgery, East Tennessee State University, Campus Box 70575, Johnson City, TN 37614-0575 USA; 6 Department of nuclear medicine, Nanjing First Hospital, Nanjing Medical University, Nanjing, 210029 China

**Keywords:** Molecular biology, Cardiovascular biology

## Abstract

Impaired fatty acid oxidation (FAO) is a prominent feature of metabolic remodeling observed in pathological myocardial hypertrophy. Hepatocyte nuclear factor 4alpha (HNF4α) is closely associated with FAO in both cellular processes and disease conditions. Pellino 1 (Peli1), an E3 ligase containing a RING-like domain, plays a crucial role in catalyzing polyubiquitination of various substrates. In this study, we aimed to investigate the involvement of HNF4α and its ubiquitination, facilitated by Peli1, in FAO during pressure overload-induced cardiac hypertrophy. Peli1 systemic knockout mice (Peli1^KO^) display improved myocardial hypertrophy and cardiac function following transverse aortic constriction (TAC). RNA-seq analysis revealed that changes in gene expression related to lipid metabolism caused by TAC were reversed in Peli1^KO^ mice. Importantly, both HNF4α and its downstream genes involved in FAO showed a significant increase in Peli1^KO^ mice. We further used the antagonist BI6015 to inhibit HNF4α and delivered rAAV9-HNF4α to elevate myocardial HNF4α level, and confirmed that HNF4α inhibits the development of cardiac hypertrophy after TAC and is essential for the enhancement of FAO mediated by Peli1 knockout. In vitro experiments using BODIPY incorporation and FAO stress assay demonstrated that HNF4α enhances FAO in cardiomyocytes stimulated with angiotension II (Ang II), while Peli1 suppresses the effect of HNF4α. Mechanistically, immunoprecipitation and mass spectrometry analyses confirmed that Peli1 binds to HNF4α via its RING-like domain and promotes HNF4α ubiquitination at residues K307 and K309. These findings shed light on the underlying mechanisms contributing to impaired FAO and offer valuable insights into a promising therapeutic strategy for addressing pathological cardiac hypertrophy.

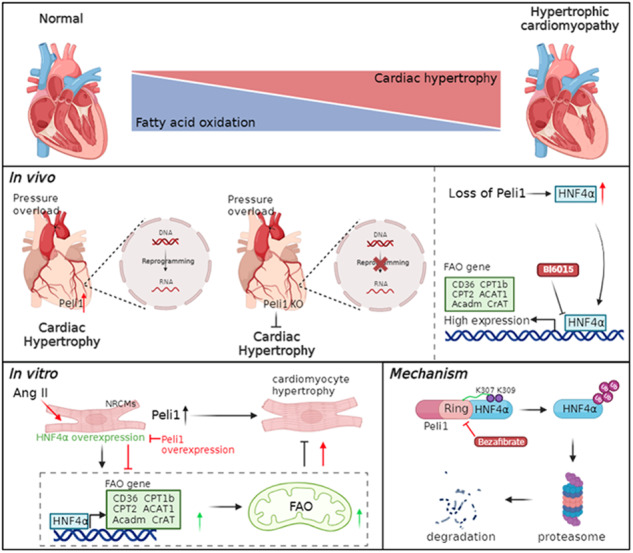

## Introduction

Cardiac hypertrophy is an adaptive response to pressure overload aimed at ensuring adequate perfusion to peripheral organs [1]. Prolonged pressure overload induces significant changes in signal transduction, metabolic processes, and mitochondrial function within the myocardium. These alterations ultimately lead to myocardial hypertrophy, systolic dysfunction, and potentially heart failure [1]. It has become increasingly evident that cardiac metabolic remodeling occurs early in the disease progression, preceding and contributing to cardiac dysfunction [2]. Consequently, there is a pressing need to explore the mechanisms underlying metabolic remodeling in cardiac hypertrophy in greater detail.

Under resting conditions, mitochondrial fatty acid oxidation (FAO) accounts for more than 70% of the adenosine 5′-triphosphate (ATP) required for cardiac energy metabolism [3]. However, the development of cardiac dysfunction resulting from pressure overload often coincides with a decrease in FAO rate [4]. These changes can be attributed to transcriptional changes in genes encoding crucial enzymes involved in FAO [5], such as carnitine palmitoyltransferase-1 (CPT-1), medium-chain acyl CoA dehydrogenase (MCAD), and cluster of differentiation 36 (CD36) [6]. Specific transcription factors like peroxisome proliferator activated receptor alpha (PPARα) [7] and peroxisome proliferative activated receptor gamma coactivator 1alpha (PGC1α) [8] frequently govern the regulation of these FAO enzymes [4]. Recent studies have emphasized the significance of maintaining FAO homeostasis to enhance cardiac function during pressure overload [2, 9, 10]. However, the precise mechanisms underlying impaired FAO during cardiac hypertrophy have not been fully elucidated.

Hepatocyte nuclear factor 4alpha (HNF4α) belongs to the nuclear receptor superfamily and plays a crucial role in hepatic lipid metabolism. Its target genes are involved in various aspects of fatty acid metabolism, including fatty acid uptake (Octn2), mitochondrial transport (CACT and CPT1), fatty acid oxidation (MCAD, VLCAD, CrAT), and others [11]. Overexpression of HNF4α has been shown to enhance FAO by increasing CES2 in nonalcoholic fatty liver disease [12]. Studies by Talianidis et al. and Michael P Verzi et al. have demonstrated that HNF4α promotes the expression of FAO genes in a fasted state and is necessary for the self-renewal of intestinal stem cells, respectively [13, 14]. The function of HNF4α in regulating FAO is influenced by changes in its protein levels and post-translational modifications [11, 15, 16]. However, the role of HNF4α and its post-translational modifications in FAO during pathological myocardial hypertrophy remains unexplored.

Pellino 1 (Peli1), a member of the Pellino protein family, is an E3 ubiquitin ligase that possesses an FHA structural domain in its N-terminus and a RING-like domain in its C-terminus. Peli1 exerts its biological functions primarily by promoting ubiquitination modifications of target proteins through its E3 ubiquitin ligase activity. For instance, Peli1 induces inflammatory cell infiltration and M1-type polarization of macrophages by promoting the ubiquitination of IRF5 in the myocardium [17]. It has also been shown to impair cardiomyocyte autophagy by promoting the ubiquitination of P62 [18]. Additionally, Peli1-mediated K48-linked polyubiquitylation and proteasome-dependent degradation of RIP3 effectively prevent cell death [19]. Recent research has identified a novel role of Peli1 in regulating the metabolic actions of T cells [20]. However, whether Peli1 can regulate metabolic processes in the myocardium is still not fully understood and requires further investigation.

In this study, we provide evidence that the absence of Peli1 confers protection against pathological cardiac hypertrophy by suppressing the ubiquitination of HNF4α and enhancing the expression of FAO-related genes in the myocardium under pressure overload conditions. These mechanisms involve the interaction between Peli1 and HNF4α through the RING-like domain, leading to the ubiquitination of HNF4α at residues K307 and K309 and subsequent degradation via the ubiquitin-proteasome system.

## Materials and methods

### Animal models

Wild-type (WT) male C57BL/6 mice and neonatal Sprague–Dawley rats were purchased from the Center of Experimental Animals, Nanjing Medical University (Nanjing, China). Peli1 systemic knockout (Peli1^KO^) mice were constructed using the Cre/LoxP system as described previously [21] and were all raised in the animal core facility of Nanjing Medical University. To generate myocardial conditional knockout Peli1 (Peli1^cKO^) mice, Peli1^Flox/Flox^ (Peli1^f/f^) mice were bred with Esr/Myh6-Cre mice. After that, tamoxifen (T-5648, Merck Millipore, Billerica, MA, USA) was injected daily for 5 days in a row at a dose of 80 mg/kg. To achieve high expression of HNF4α specifically in mouse myocardium, six-week-old WT male C57BL/6 mice were employed and randomly injected with 200 μL of saline containing 3 × 10^11^ vector genomes (vg) of recombinant adeno-associated virus 9 (rAAV9)-GFP or rAAV9-HNF4α vectors via the tail vein respectively, and GFP castle was inserted for efficiency detection. After injecting for 2 weeks, animals were subjected to transverse aortic constriction (TAC). These animals were kept under constant environmental conditions with 12 h of light/dark cycles, with free access to food and water. Animal experiments were performed in accordance with the Guide for the Care and Use of Laboratory Animals published by the National Institutes of Health (NIH Publication No. 85-23, revised 1996) and were approved by the Nanjing Medical University Committee on Animal Care (Approval NO. IACUC-1811028).

Male mice (8 weeks, 22–25 g) were subcutaneously (s.c.) pre-treated with 2 mg/kg butorphanol (Jiangsu Hengrui Pharmaceuticals Co.). Anesthesia was induced with 5% isoflurance (R510-22-10, RWD Life Science, Shenzhen, China) in an induction chamber. After oral intubation, mice maintained anesthesia with 1.5% isoflurane and placed on a surgical table which was temperature-controlled. The aortic arch was exposed and a 0.5 mm-wire was placed alongside the transverse aorta. The wire was ligated with artery by the 5–0 silk and immediately removed, leaving the aortic arch constricted to the diameter of the wire. For the sham operation, the identical procedure was performed without the tying of the suture around the aorta. Peli1^KO^ mice that underwent TAC surgery were injected intraperitoneally with vehicle (5% DMSO, D8372, Solarbio, Beijing, China) or BI6015 (10 mg/kg/d, E0401, Selleck Chemicals, Houston, TX, USA) in a volume of 100 μL once daily for 28 days continuously. At last, mice were killed by cervical dislocation after an overdose of 5% isoflurane. During the experiment, the operator was not aware of the animals’ grouping and genotype. There was no statistical procedure employed to calculate the sample size for the mouse experiment, which was relied on preliminary experimental data. The sample size for each experiment is provided in the legend.

### Culture and treatment of NRCMs

Neonatal rat ventricular myocytes (NRCMs) were isolated from 1 to 3-day-old neonatal Sprague–Dawley rats as previously described [22], NRCMs were plated at a density of 1 × 10^6^ cells/mL and cultured in Dulbecco’s modified Eagle’s medium (DMEM, Gibco, Grand Island, NY, USA) supplemented with penicillin (Beyotime Biotechnology, Shanghai, China), streptomycin (Beyotime Biotechnology, Shanghai, China), 10% fetal bovine serum (FBS, Biological Industries, Kibbutz Beit-Haemek, Israel), 10 mM HEPES, and 0.1 mM 5-Bromo-2′-deoxyuridine (BrdU, Sigma-Aldrich, St. Louis, MO, USA). 36 h after the NRCMs were seeded, the adherent cells were infected with adv-Peli1 (MOI = 50) and adv-HNF4α (MOI = 50). Four hours later, the culture medium containing the virus was replaced with fresh medium. After 24 h, NRCMs were treated with angiotension II (Ang II, Sigma-Aldrich, St. Louis, MO, USA) at a concentration of 1 μM for 24 h. In addition, NRCMs transduced with adv-HNF4α (MOI = 50), adv-Peli1 (MOI = 50) or adv-GFP (MOI = 50) for 48 h, followed by treatment with 100 μM cycloheximide (HY-12320, MedChem Express, Monmouth Junction, NJ, USA) for 0.5, 1, 2 and 4 h to detect the degradation levels of HNF4α.

### Histology

Heart samples acquired from each group were fixed in 4% polyformaldehyde solution. After 24 h later, they were embedded in paraffin (A6330, Millipore, Billerica, MA, USA), cut at 5 μm. The tissues were counter stained with hematoxylin and eosin (H&E, Solarbio, Beijing, China), and examined with bright field microscopy (BX51, OLYMPUS, Tokyo, Japan) to detect the cross-sectional area of cardiomyocytes. Additionally, the tissues were labeled with WGA (W11261, Thermo Fisher Scientific, Waltham, MA, USA) staining, incubated at 37 °C in the dark for 30 min. The fluorescence detection was performed under the fluorescence microscope (AX10, Zeiss, German).

### Echocardiographic analyses

The evaluation of cardiac functions was used by Echocardiography (GE Vivid 7equipped with a 14-MHz phase array linear transducer, S12, allowing a 150 maximal sweep rate). The assessment for the extent of PO was determined via measuring the right common carotid artery/left common carotid artery flow ratio (RCCA/LCCA) within one week after the surgery and only mice with a ratio of 7–10 were used for the experiments. The mice were anesthetized by a mixture of isoflurane inhalation (1.5%) and oxygen (0.5 L/min), and the body temperature was maintained at 37 °C on a heating table. M-mode tracings were used to measure interventricular septum diastolic dimension (IVS), left ventricular posterior wall diastolic dimension (LVPW), and left ventricular internal dimension diastole (LVID). Percent ejection fraction (EF%) and percent fractional shortening (FS%) were calculated as described in our previous study [23, 24]. All measurements were averaged over six consecutive cardiac cycles and made by the observer who was blinded concerning the identity of the tracings.

### Western blot

Left ventricular myocardial tissue or cardiomyocytes were isolated and lysed with cold 1 × lysis buffer supplemented with a protease inhibitor cocktail (Thermo Fisher Scientific, Waltham, MA, USA). Protein concentration was measured using a BCA protein assay kit (Thermo Fisher Scientific, Waltham, MA, USA) and then mixed with 1 × SDS sample buffer and boiled at 95 °C for 5 min. The lysate was resolved by SDS-PAGE and transferred onto polyvinylidene difluoride (PVDF) membranes, which were blocked and incubated with primary antibodies at 4 °C overnight, followed by incubation with horseradish peroxidase (HRP)-conjugated secondary antibodies. The signals were detected using the ECL system (Pierce) and quantified by scanning densitometry using the Image Lab analysis system (see Supplementary Material for original western blots). The membranes were stripped with stripping buffer (P0025, Beyotime, Beijing, China) and reblotted with next primary antibody. Primary antibodies: anti-Peli1 (ab199336, Abcam, Cambridge, MA, USA), anti-HNF4α (ab41898, Abcam, Cambridge, MA, USA), anti-GAPDH (AF0006, Beyotime, Beijing, China), anti-Flag (F2555, Sigma-Aldrich, St. Louis, MO, USA), anti-Myc (AF2864, Beyotime, Beijing, China), anti-His (AF2876, Beyotime, Beijing, China), anti-HA (AF2858, Beyotime, Beijing, China).

### Quantitative real-time PCR (qRT-PCR)

As for mRNA detection, the cells and tissues were lysed by Total RNA Extraction Reagent (TRIzol Reagent, R401-01-AA, Vazyme Biotech, Nanjing, China) and then total RNA was extracted. cDNA was generated by using PrimeScript^TM^ RT Reagent kit with gDNA eraser (RR047A, Takara Biomedical Technology, Tokyo, Japan). QPCR was performed on QuantStudio (TM) 6 Flex System (Applied Biosystems, Thermo Fisher Scientific, Waltham, MA, USA) via using ChamQ^TM^ SYBR Color qPCR Master Mix (Q411-02, Vazyme Biotech, Nanjing, China), with every sample prepared in triplicate following the recommendation of the manufacturer and hypoxanthine-guanine phosphoribosyltransferase (HPRT) as the internal reference. The relative expression level of mRNAs was measured by using 2^-ΔΔCT^method. The following are the primer sequences:

List of relative primers

**Table:** 

Gene	Forward	Reverse
Mus-HPRT	GTTGGATACAGGCCAGACTTTGTT	GATTCAACTTGCGCTCATCTTAGGC
Mus-ANP	GAGAAGATGCCGGTAGAAGA	AAGCACTGCCGTCTCTCAGA
Mus-BNP	CTGCTGGAGCTGATAAGAGA	TGCCCAAAGCAGCTTGAGAT
Mus-β-MHC	GTGCCAAGGGCCTGAATGAG	GCAAAGGCTCCAG-GTCTGA
Mus-CD36	AAAGTTGCCATAATTGAGTCCT	TCCGAACACAGCGTAGATAGA
Mus-CPT1b	TGCCTTTACATCGTCTCCAA	AGACCCCGTAGCCATCATC
Mus-CPT2	AGCCAGTTCAGGAAGACAGA	GACAGAGTCTCGAGCAGTTA
Mus-CrAT	GCTGCCAGAACCGTGGTAAA	CCTTGAGGTAATAGTCCAGGGA
Mus-ACAT1	CAGGAAGTAAGATGCCTGGAAC	TTCACCCCCTTGGATGACATT
Mus-Acadm	AGGGTTTAGTTTTGAGTTGACGG	CCCCGCTTTTGTCATATTCCG
Rat-HPRT	GCTGAAGATTTGGAAAAGGTGT	ACAGAGGGCCACAATGTGAT
Rat-ANP	CACCAAGGGCTTCTTCCTC	CGAGAGCACCTCCATCTCTC
Rat-BNP	CGGGCTGAGGTTGTTTTAGG	GCCGCAGGCAGAGTCAGA
Rat-β-MHC	GCCCCAAATGCAGCCAT	CGCTCAGTCATGGCGGAT
Rat-CD36	CCTCGGATGGCTAGCTGATT	TGTGGCCTGGTTCAACTAAT
Rat-CPT1b	AGTGTGCCAGCCACAATTCA	ATAGGCTTCGTCATCCAGCAA
Rat-CPT2	CTAAGAGATGCTCCGAGGCG	TCAAAGCCCTGGCCCATCG
Rat-CrAT	CTTTCTACCAGCCAGGTCCC	TAGCAGATGCCGTAACCGTC
Rat-ACAT1	GCCATCCAATCGGGATGTCT	AGTTCACTTCAGCGGGTCAC
Rat-Acadm	GGGGAAAGGCCAACTGGTAT	AGCCCCCATTGCAATCTTGA

### RNA sequencing

Total RNA was extracted from mice left ventricular myocardial tissues using Total RNA Extraction Reagent (TRIzol Reagent, R401-01-AA, Vazyme Biotech, Nanjing, China). RNA sequencing was assigned to BGI Genomics for sequencing analysis using the Illumina HiSeq. All raw data were filtered using SOAPnuke and later compared with the GRCm38.p5 genome using Hierarchical Indexing for Spliced Alignment of Transcripts (HISAT) software. Clustering analysis was then performed using the Dr.TOM website of BGI Genomics (https://biosvs.bgi.com).

### Palmitate oxidation stress test

The experiment was performed according to fatty acid oxidative stress test kit (103693, Agilent, Technologies, Santa Clara, CA, USA) instructions. NRCMs were adherent in an Agilent Seahorse XF96 Cell Culture Microplate, treated with adv-GFP or adv-HNF4α (MOI: 50) for 24 h, and then treated with Ang II (1 × 10^−6 ^mol/L) for 24 h. The medium in the microplates was replaced with 100 μL of Substrate-Limited Growth Media and incubated overnight. Later, replace with 180 μL of Substrate-Limited Assay Media and incubate in a CO_2_-free incubator for 60 min. Add palmitate-BSA to the corresponding cell plate wells. Add 20 μL of oligo, FCCP, and AA/ROT working solutions to the probe plate to give final well concentrations of 2 μM, 40 μM, and 2 μM/2 μM, respectively. Put the probe plate into the analyzer for calibration and put it into the cell plate for detection after completion.

### Fatty acid analog uptake

NRCMs were seeded in a 24-well plates, treated with adv-GFP or adv-HNF4α (MOI: 50) for 24 h, and then treated with Ang II (1 × 10^−6 ^mol/L) for 24 h. Next, the cells were incubated for 30 min with 5 μM BODIPY™ 558/568 C12 (4,4-difluoro-5-(2-thienyl)-4-bora-3a,4a-diaza-s-indacene-3-dodecanoic acid, D3835, Thermo Fisher Scientific, Waltham, MA, USA) in DMEM and observed by fluorescence microscopy. After that, cells were treated with DMEM without BODIPY™ 558/568 C12 for 5.5 h and observed by fluorescence microscopy. For the experimental procedure, see Fig. 5f.

### Co-immunoprecipitation (Co-IP)

Lysates were incubated with antibodies for 4 h at 4 °C on a rotator, followed by overnight incubation with pre-cleared protein A/G agarose (Thermo Fisher Scientific, Waltham, MA, USA) overnight at 4 °C under rotation. The samples were centrifuged and washed thrice with washing buffer supplemented with 500 mM NaCl and then mixed with 2× SDS sample buffer and boiled at 95 °C for 5 min. The precipitates were resolved by SDS-PAGE and transferred onto polyvinylidene difluoride (PVDF) membranes, which were blocked and incubated with primary antibodies at 4 °C overnight, followed by incubation with horseradish peroxidase (HRP)-conjugated secondary antibodies. The signals were detected using the ECL system (Pierce) and quantified by scanning densitometry using the Image Lab analysis system.

### Bimolecular affinity purification and liquid chromatography-mass spectrometry (LC-MS)

HEK293T cells were purchased from National Collection of Authenticated Cell Cultures (NCACC, Shanghai, China) and were cultured with DMEM (Gibco, Grand Island, NY, USA) supplemented with 10% FBS (Biological Industries, Kibbutz Beit-Haemek, Israel). The pEBB-TB-HNF4α plasmid, His-Ub plasmid and myc-Peli1 plasmid were transfected in HEK293T cells. Bimolecular labeled ubiquitination modified HNF4α complexes in the cell lysates were captured by using streptavidin-agarose column (20347, Thermo Fisher Scientific, Waltham, MA, USA) and nickel-agarose column (P2226, Beyotime, Beijing, China) [25]. The protein complexes were analyzed by SDS-PAGE after elution. The gels were stained with Coomassie blue, and the target bands were excised for liquid chromatography-mass spectrometry.

### Statistical analysis

GraphPad Prism 7.00 (GraphPad Software) was used for statistical analysis. Results were presented as mean ± SD. All comparisons between 2 groups were performed with two-tailed unpaired Student’s *t*-test in the condition of homogeneity of variance (*p* > 0.1). If not, we used unpaired *t*-test with Welch’s correction. One-way ANOVA analysis, with Tukey’s post hoc test, was used for comparing among ≥3 groups. *p* < 0.05 was considered statistically significant.

## Results

### Peli1 deficiency ameliorates pressure overload-induced cardiac hypertrophy

**Fig. 1:**
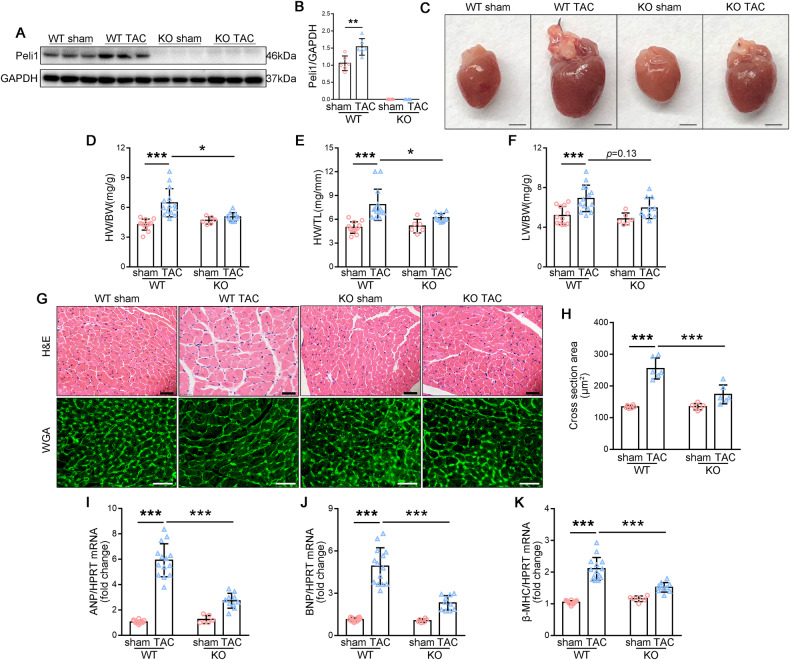
Peli1 knockout ameliorates TAC-induced cardiac hypertrophy. 8-week-old WT and Peli1^KO^ mice were randomly subjected to transverse aortic constriction (TAC) or sham surgery for 4 weeks. **A**, **B** Western blot analysis of Peli1 in the left ventricular myocardium, GAPDH as the loading control. (*n* = 6). **C** Representative gross morphology of hearts (*n* = 12 for WT sham group, *n* = 14 for WT TAC group, *n* = 6 for KO sham group and *n* = 10 for KO TAC group). Scale bar: 2 mm. **D**–**F** Heart weight-to-body weight (HW/BW) ratio (**D**), heart weight-to-tibia length (HW/TL) ratio (**E**), and lung weight-to-body weight (LW/BW) ratio (**F**) (*n* = 12 for WT sham group, *n* = 14 for WT TAC group, *n* = 6 for KO sham group and *n* = 10 for KO TAC group). **G** Representative graphs of H&E staining (top) and WGA staining (bottom). (*n* = 6). Scale bar: 40 μm. **H** Cross sectional area of cardiomyocytes counted by WGA staining. (*n* = 6). **I**–**K** qRT-PCR was performed to analyze the mRNA levels of atrial natriuretic peptide (ANP) (**I**), brain natriuretic peptide (BNP) (**J**), and myosin heavy chain beta (β-MHC) (**K**) (*n* = 12 for WT sham group, *n* = 14 for WT TAC group, *n* = 6 for KO sham group and *n* = 10 for KO TAC group). Values are mean ± SD. *p < 0.05, **p < 0.01, ***p < 0.001 (one-way ANOVA followed by Tukey’s test).

To investigate the potential role of Peli1 during cardiac hypertrophy in vivo, we conducted TAC surgery on WT mice and Peli1^KO^ mice for 4 weeks. TAC surgery prominently increased the expression of Peli1 in the left ventricular myocardial tissue of WT mice, while it was undetectable in the Peli1^KO^ groups (Fig. 1A, B). Notably, TAC-induced cardiac hypertrophy observed in WT mice was significantly inhibited by the absence of Peli1 (Fig. 1C). Consistently, this effect was also reflected in the heart weight-to-body weight (HW/BW) ratio, heart weight-to-tibia length (HW/TL) ratio, and lung weight-to-body weight (LW/BW) ratio (Fig. 1D–F). Histological examinations using H&E and WGA staining further revealed that Peli1 knockout markedly reduced the cross-sectional area of cardiomyocytes compared to WT mice after TAC (Fig. 1G, H). Moreover, the up-regulation of myocardial hypertrophy biomarkers including atrial natriuretic peptide (ANP), brain natriuretic peptide (BNP), and myosin heavy chain beta (β-MHC) mRNA levels which induced by TAC were significantly attenuated in Peli1-deficient hearts (Fig. 1I–K). These findings indicate that Peli1 is involved in the development of cardiac hypertrophy following TAC and its absence ameliorates TAC-induced myocardial hypertrophy.

Echocardiography analysis of cardiac function demonstrated that WT mice following TAC exhibited a notable decrease in EF% and FS%, along with an increase in IVS;d, IVS;s, LVID;d, LVID;s, LVPW;d, and LVPW;s. In contrast, Peli1^KO^ mice displayed higher EF% and FS%, as well as lower IVS;d, IVS;s, LVID;d, LVID;s, LVPW;d, and LVPW;s compared to WT mice following TAC (Table 1). These results indicate that Peli1 knockout improves TAC-induced cardiac dysfunction.

**Table 1:** Peli1 deficiency ameliorates TAC-induced cardiac dysfunction.

Groups	WT Sham	WT TAC	Peli1^KO^ Sham	Peli1^KO^ TAC
*n*	12	14	6	10
EF (%)	61.540 ± 2.793	43.516 ± 8.818***	60.365 ± 3.096	54.257 ± 2.690^###^
FS (%)	32.321 ± 1.843	24.265 ± 5.236***	31.630 ± 2.046	28.046 ± 1.455^#^
Ivs;d (mm)	0.710 ± 0.024	0.994 ± 0.133***	0.731 ± 0.043	0.914 ± 0.070^#^
Ivs;s (mm)	1.141 ± 0.072	1.349 ± 0.143***	1.146 ± 0.092	1.279 ± 0.092 ^*p* = 0.19^
LVID;d (mm)	3.562 ± 0.187	3.980 ± 0.396**	3.745 ± 0.229	3.873 ± 0.238^#^
LVID;s (mm)	2.413 ± 0.185	3.028 ± 0.184***	2.563 ± 0.220	2.831 ± 0.223^#^
LVPW;d (mm)	0.734 ± 0.401	1.057 ± 0.174***	0.758 ± 0.031	0.968 ± 0.113^#^
LVPW;s (mm)	1.193 ± 0.070	1.415 ± 0.163***	1.206 ± 0.072	1.332 ± 0.089^#^

Cardiac function was measured by echocardiography at 4 weeks after TAC. Data are shown as mean ± SD. Statistical analysis was performed with one-way ANOVA followed by Tukey’s test.

**p < 0.01; ***p < 0.001 vs WT Sham group.

^#^*p* < 0.05; ^###^*p* < 0.001; *p* = 0.19 (not significant) vs WT TAC group.

### Peli1 deletion causes a reprogramming of fatty acid metabolic pathways in pathological myocardial hypertrophy

**Fig. 2:**
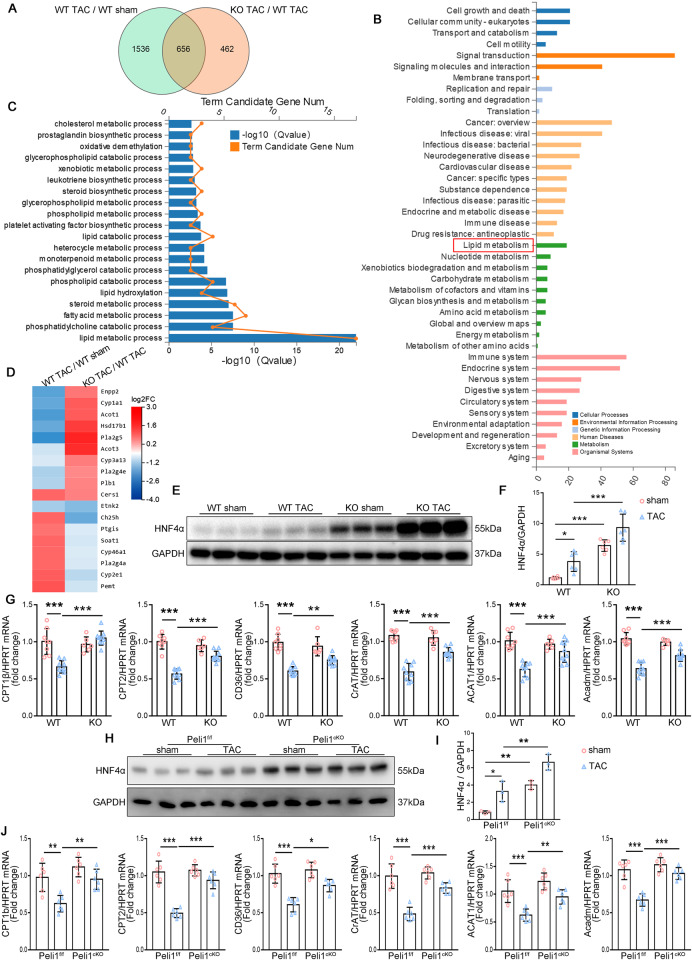
Peli1 deletion alters TAC-induced changes in fatty acid gene expression profiles. **A**–**G** 8-week-old WT and Peli1^KO^ mice were randomly subjected to TAC or sham surgery for 4 weeks. RNA-seq analysis was performed using mRNA extracted from left ventricular myocardium. **A** Venn diagram of differentially expressed genes that changed more than 2-fold in the WT TAC group compared to the WT sham group and KO TAC group compared to the WT TAC group (*n* = 3). **B** Kyoto Encyclopedia of Genes and Genomes (KEGG) enrichment analysis of these 656 genes. **C** Line graph of GO_ Biological Process (GO_P) analysis of genes under the category “lipid metabolic process” (red rectangle) in (**B**). **D** Heat map showing the variation of differentially expressed genes under the catabolic categories in (**C**). **E**, **F** Western blot analysis of protein levels of HNF4α, GAPDH as the loading control (*n* = 6). **G** qRT-PCR was performed to analyze the mRNA levels of CPT1b, CPT2, CD36, CrAT, ACAT1 and Acadm (*n* = 8 for WT sham group, *n* = 9 for WT TAC group, *n* = 6 for KO sham group and *n* = 10 for KO TAC group). **H**–**J** 8-week-old Peli1^f/f^ and Peli1^cKO^ mice were subjected to TAC or sham surgery for 4 weeks. **H**, **I** Western blot analysis of protein levels of HNF4α, GAPDH as the loading control (*n* = 3). **J** qRT-PCR was performed to analyze the mRNA levels of CPT1b, CPT2, CD36, CrAT, ACAT1 and Acadm (*n* = 6). Values are mean ± SD. *p < 0.05, **p < 0.01, ***p < 0.001 (one-way ANOVA followed by Tukey’s test).

To investigate the progressive changes following Peli1 knockout, we conducted RNA sequencing (RNA-seq) analysis to compare mRNA expression profiles in the hearts of WT and Peli1^KO^ mice at 4 weeks after TAC. We observed differential expression of 2192 genes that changed more than 2-fold in WT mice in response to TAC, while 1118 genes showed more than 2-fold differential expression in the Peli1^KO^ TAC group compared to the WT TAC group. Interestingly, 656 genes overlapped between these two sets of differentially expressed genes (Fig. 2A). Kyoto Encyclopedia of Genes and Genomes (KEGG) enrichment analysis of these 656 genes revealed a notable impact of Peli1 on the transcription of “metabolism”, with “lipid metabolism” being the most pronounced (highlighted by the red rectangle) (Fig. 2B). Further examination through GO enrichment analysis of genes involved in the “lipid metabolism” category revealed that Peli1 influenced the transcription of genes related to “lipid metabolic process” (Fig. 2C). The relative expression of Ch25h, Ptgis, Soat1, Cyp46a1, and others increased after TAC, whereas it decreased dramatically by Peli1 knockout (Fig. 2D). However, the relative expression heat map of some “lipid metabolic process” genes, such as Enpp2, Cyp1a1, Acot1, et al., including genes involved in FAO, demonstrated a significant decrease following TAC, while their expression rebounded significantly after Peli1 knockout (Fig. 2D). These findings collectively indicate that Peli1 deletion affects the transcription of myocardial lipid catabolism genes following TAC.

Since it has been shown that genes involved in FAO are commonly decreased after pressure overload, we concentrated on genes in the upper half of the heat map. Further analysis using the rVista2.0 database revealed that most of these genes possess binding sites for HNF4α in their transcriptional regulatory regions (Supplementary Table 1). Therefore, we examined the expression of HNF4α in TAC-induced myocardial hypertrophy. We observed significant increases in HNF4α levels in both WT and Peli1^KO^ mice after TAC compared to the sham groups. Moreover, Peli1^KO^ mice exhibited a more robust increase in HNF4α expression following TAC compared to WT mice (Fig. 2E, F). To determine whether the reprogramming of fatty acid metabolic pathways in Peli1 knockout is associated with changes in HNF4α abundance, we performed qRT-PCR analysis to examine the expression of HNF4α target genes involved in FAO, including CPT1b, CPT2, CD36, CrAT, ACAT1, and Acadm (coding for MCAD). We observed that these genes were downregulated by TAC surgery but exhibited increased expression in the hearts of Peli1^KO^ mice (Fig. 2G). These results suggest that the elevated level of HNF4α resulting from Peli1 deletion might impact the transcription of FAO-related genes.

Further studies were performed in myocardial conditional knockout Peli1 (Peli1^cKO^) mice. Peli1^Flox/Flox^ (Peli1^f/f^) mice and Peli1^cKO^ mice were subjected to sham or TAC surgery, and HNF4α protein expression in left ventricular myocardial tissue was examined after 4 weeks. Conditional knockout Peli1 in myocardial similarly caused a significant increase in HNF4α (Fig. 2H, I). Similar to Peli1^KO^ mice, Peli1^cKO^ mice showed a marked increase in the expression of genes involved in FAO after TAC (Fig. 2J). These results indicate that Peli1 may play a regulatory role in FAO in myocardium by regulating HNF4α expression.

### Inhibition of HNF4α suppresses the ameliorative effect of Peli1 knockout on pathological cardiac hypertrophy

**Fig. 3:**
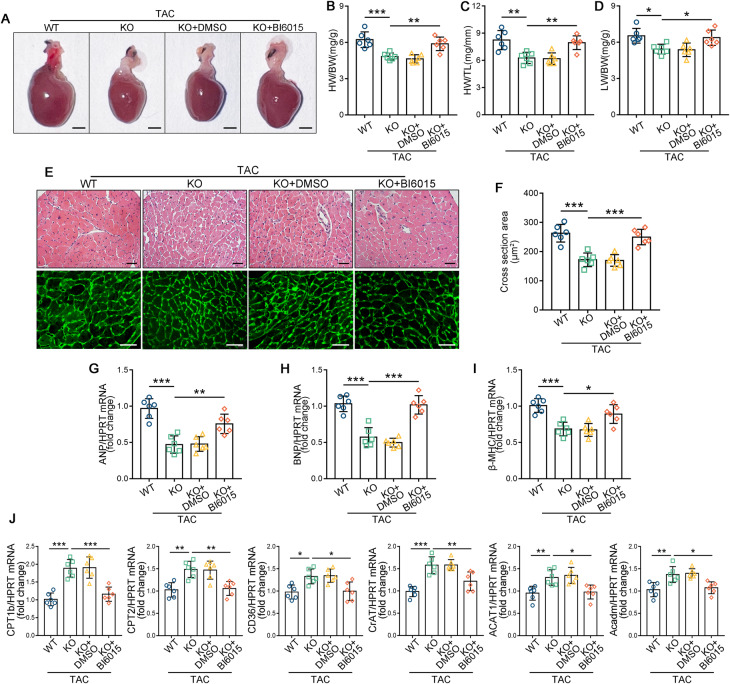
HNF4α antagonist BI6015 inhibits the ameliorative effect of Peli1 deficiency on pathological myocardial hypertrophy. 8-week-old WT and Peli1^KO^ mice were randomly subjected to TAC surgery, Peli1^KO^ mice were continuously administered intraperitoneally with BI6015 or 5% DMSO for 4 weeks postoperatively. **A** Representative gross morphology of hearts (*n* = 6). Scale bar: 2 mm. **B**–**D** HW/BW ratio (**B**), HW/TL ratio (**C**), and LW/BW ratio (**D**) (*n* = 6). **E** Representative graphs of H&E staining (top) and WGA staining (bottom) (*n* = 6). Scale bar: 40 μm. **F** Cross sectional area of cardiomyocytes counted by WGA staining. **G**–**I** qRT-PCR was performed to analyze the mRNA levels of ANP (**G**), BNP (**H**) and β-MHC (**I**) (*n* = 6). **J** qRT-PCR was performed to analyze the mRNA levels of CPT1b, CPT2, CD36, CrAT, ACAT1 and Acadm (*n* = 6). Values are mean ± SD. *p < 0.05, **p < 0.01, ***p < 0.001 (one-way ANOVA followed by Tukey’s test).

To validate whether the beneficial effect of Peli1 knockout on cardiac hypertrophy relies on HNF4α, we utilized the HNF4α antagonist BI6015 [26]. Consistent with previous studies, Peli1 knockout significantly improved TAC-induced cardiac hypertrophy, but this was significantly suppressed by BI6015 administration (Fig. 3A). Analysis of histological data revealed that the ratios of HW/BW, HW/TL, and LW/BW were significantly reduced in Peli1^KO^ mice compared to WT mice after TAC, while the administration of BI6015 inhibited these changes (Fig. 3B–D). H&E staining and WGA staining demonstrated that BI6015 administration led to significantly larger cardiomyocytes compared to the Peli1^KO^ TAC group (Fig. 3E, F). QRT-PCR analysis revealed that BI6015 administration also significantly upregulated the mRNA levels of ANP, BNP, and β-MHC in Peli1^KO^ mice after TAC (Fig. 3G–I). Echocardiography examination showed that Peli1 knockout notably ameliorated TAC-induced cardiac dysfunction, as evidenced by a significant increase in EF% and FS%, and a decrease in IVS, LVPW, and LVID. However, Peli1^KO^ mice treated with BI6015 exhibited a significant reduction in these beneficial effects (Table 2). These data suggest that HNF4α is involved in the ameliorative effect of Peli1 knockout on TAC-induced pathological myocardial hypertrophy. Furthermore, these changes were accompanied by alterations in the transcription of FAO genes (CD36, CPT1b, CPT2, CrAT, ACAT1, Acadm) which were significantly decreased in Peli1^KO^ mice treated with BI6015 after TAC (Fig. 3J). These results indicate that the promotion of FAO gene transcription and the amelioration of cardiac hypertrophy were dependent on HNF4α.

**Table 2:** HNF4α antagonist BI6015 inhibits the ameliorative effect of Peli1 knockout on TAC-induced cardiac dysfunction.

Groups	WT TAC	Peli1^KO^ TAC	Peli1^KO^ TAC + DMSO	Peli1^KO^ TAC + BI6015
*n*	6	6	6	6
EF (%)	41.325 ± 3.335	53.623 ± 3.422***	53.175 ± 3.861	45.162 ± 2.907^##^
FS (%)	20.063 ± 1.798	27.257 ± 2.221***	27.025 ± 2.545	22.197 ± 1.599^##^
Ivs;d (mm)	1.013 ± 0.068	0.751 ± 0.107**	0.767 ± 0.130	0.981 ± 0.135^#^
Ivs;s (mm)	1.330 ± 0.049	1.076 ± 0.089**	1.104 ± 0.0169	1.320 ± 0.147^#^
LVID;d (mm)	4.326 ± 0.278	3.678 ± 0.288**	3.789 ± 0.234	4.169 ± 0.286^#^
LVID;s (mm)	3.461 ± 0.279	2.675 ± 0.216***	2.764 ± 0.183	3.246 ± 0.275^##^
LVPW;d (mm)	1.094 ± 0.156	0.778 ± 0.137*	0.795 ± 0.195	1.055 ± 0.131^#^
LVPW;s (mm)	1.200 ± 0.283	1.232 ± 0.289	1.204 ± 0.282	1.193 ± 0.151^#^

Cardiac function was measured by echocardiography at 4 weeks after TAC. Data are shown as mean ± SD. Statistical analysis was performed with one-way ANOVA followed by Tukey’s test.

*p < 0.05; **p < 0.01; ***p < 0.001 vs WT TAC group.

^#^*p* < 0.05; ^##^*p* < 0.01 vs Peli1^KO^ TAC group.

### Myocardial-specific HNF4α overexpression represses TAC-induced cardiac hypertrophy and fatty acid metabolism disorder

**Fig. 4:**
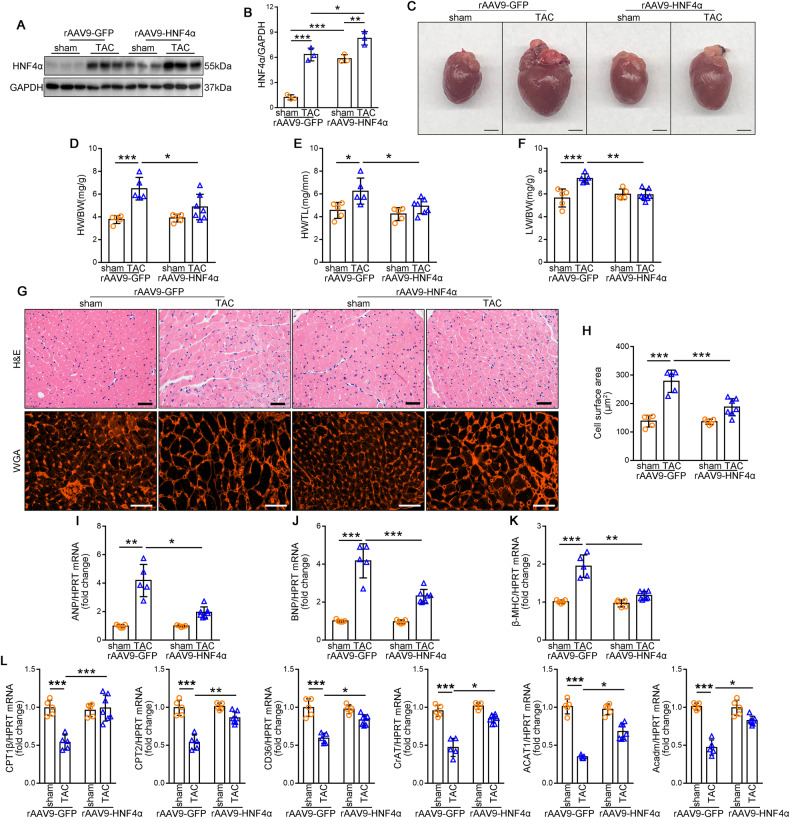
Cardiac-specific HNF4α overexpression represses TAC-induced cardiac hypertrophy and fatty acid metabolism disorder. 6-week-old, male mice were injected with rAAV9-HNF4α or rAAV9-GFP (3 × 10^11^ vg) via tail-vein injection for 2 weeks and then randomly subjected to TAC or sham surgery for 4 weeks. **A**, **B** Western blot analysis of protein levels of HNF4α, GAPDH as the loading control (*n* = 3). **C** Representative gross morphology of hearts (*n* = 5 for rAAV9-GFP sham group, *n* = 5 for rAAV9-GFP TAC group, *n* = 5 for rAAV9-HNF4α sham group and *n* = 7 for rAAV9-HNF4α TAC group). Scale bar: 2 mm. **D**–**F** HW/BW ratio (**D**), HW/TL ratio (**E**), and LW/BW ratio (**F**) (*n* = 5 for rAAV9-GFP sham group, *n* = 5 for rAAV9-GFP TAC group, *n* = 5 for rAAV9-HNF4α sham group and *n* = 7 for rAAV9-HNF4α TAC group). **G** Representative graphs of H&E staining (top) and WGA staining (bottom) (*n* = 5 for rAAV9-GFP sham group, *n* = 5 for rAAV9-GFP TAC group, *n* = 5 for rAAV9-HNF4α sham group and *n* = 7 for rAAV9-HNF4α TAC group). Scale bar: 40 μm. **H** Cross sectional area of cardiomyocytes counted by WGA staining (*n* = 5 for rAAV9-GFP sham group, *n* = 5 for rAAV9-GFP TAC group, *n* = 5 for rAAV9-HNF4α sham group and *n* = 7 for rAAV9-HNF4α TAC group). **I**–**K** qRT-PCR was performed to analyze the mRNA levels of ANP (**I**), BNP (**J**) and β-MHC (**K**) (*n* = 5 for rAAV9-GFP sham group, *n* = 5 for rAAV9-GFP TAC group, *n* = 5 for rAAV9-HNF4α sham group and *n* = 7 for rAAV9-HNF4α TAC group). **L** qRT-PCR was performed to analyze the mRNA levels of CPT1b, CPT2, CD36, CrAT, ACAT1 and Acadm (*n* = 5 for rAAV9-GFP sham group, *n* = 5 for rAAV9-GFP TAC group, *n* = 5 for rAAV9-HNF4α sham group and *n* = 7 for rAAV9-HNF4α TAC group). Values are mean ± SD. *p < 0.05, **p < 0.01, ***p < 0.001 (one-way ANOVA followed by Tukey’s test).

Furthermore, it is crucial to evaluate whether HNF4α overexpression in the heart can rescue cardiac hypertrophy and normalize FAO genes in vivo. For this purpose, rAAV9 vectors were administered via tail-vein injection to infect the heart. The introduction of rAAV9-HNF4α significantly increased the expression of HNF4α in the heart (Fig. 4A, B) and attenuated TAC-induced cardiac hypertrophy (Fig. 4C). Consistently, the HW/BW, HW/TL, and LW/BW ratios were decreased in the rAAV9-HNF4α TAC group compared to the rAAV9-GFP TAC group (Fig. 4D–F). Cardiac-specific overexpression of HNF4α rescued the TAC-induced increase in cardiomyocyte cross-sectional area, as observed in H&E and WGA staining (Fig. 4G, H). Additionally, qRT-PCR analysis demonstrated a significant reduction in the transcription of ANP, BNP, and β-MHC in AAV9-HNF4α mice after TAC (Fig. 4I–K). Echocardiography analyses revealed a significantly improved cardiac function in AAV9-HNF4α mice compared to rAAV9-GFP mice after TAC, as indicated by increased EF% and FS% (Table 3). Importantly, cardiac overexpression of HNF4α significantly upregulated the expression of FAO genes (CPT1b, CPT2, CD36, CrAT, ACAT1, and Acadm) after TAC (Fig. 4L).

**Table 3:** Overexpression of HNF4α ameliorates TAC-induced cardiac dysfunction.

Groups	rAAV9-GFP Sham	rAAV9-GFP TAC	rAAV9-HNF4α Sham	rAAV9-HNF4α TAC
*n*	5	5	5	6
EF (%)	58.817 ± 2.123	44.453 ± 4.030***	57.877 ± 2.329	54.791 ± 7.330^##^
FS (%)	30.638 ± 1.556	21.911 ± 2.365**	29.482 ± 1.011	28.094 ± 4.699^#^
Ivs;d (mm)	0.723 ± 0.360	0.966 ± 0.086***	0.720 ± 0.037	0.774 ± 0.026^###^
Ivs;s (mm)	1.172 ± 0.080	1.274 ± 0.038**	1.050 ± 0.060	1.129 ± 0.043^###^
LVID;d (mm)	3.577 ± 0.216	4.227 ± 0.227***	3.665 ± 0.149	3.900 ± 0.153^#^
LVID;s (mm)	2.686 ± 0.099	3.301 ± 0.216***	2.584 ± 0.101	2.803 ± 0.200^###^
LVPW;d (mm)	0.746 ± 0.036	1.008 ± 0.050***	0.745 ± 0.035	0.832 ± 0.040^###^
LVPW;s (mm)	1.241 ± 0.033	1.393 ± 0.578**	1.084 ± 0.084	1.266 ± 0.072^#^

Cardiac function was measured by echocardiography at 4 weeks after TAC. Data are shown as mean ± SD. Statistical analysis was performed with one-way ANOVA followed by Tukey’s test.

**p < 0.01; ***p < 0.001 vs rAAV9-GFP Sham group.

^#^*p* < 0.05; ^##^*p* < 0.01; ^###^*p* < 0.001 vs rAAV9-GFP TAC group.

### Peli1 inhibits the improving effect of HNF4α on FAO in cardiomyocytes

**Fig. 5:**
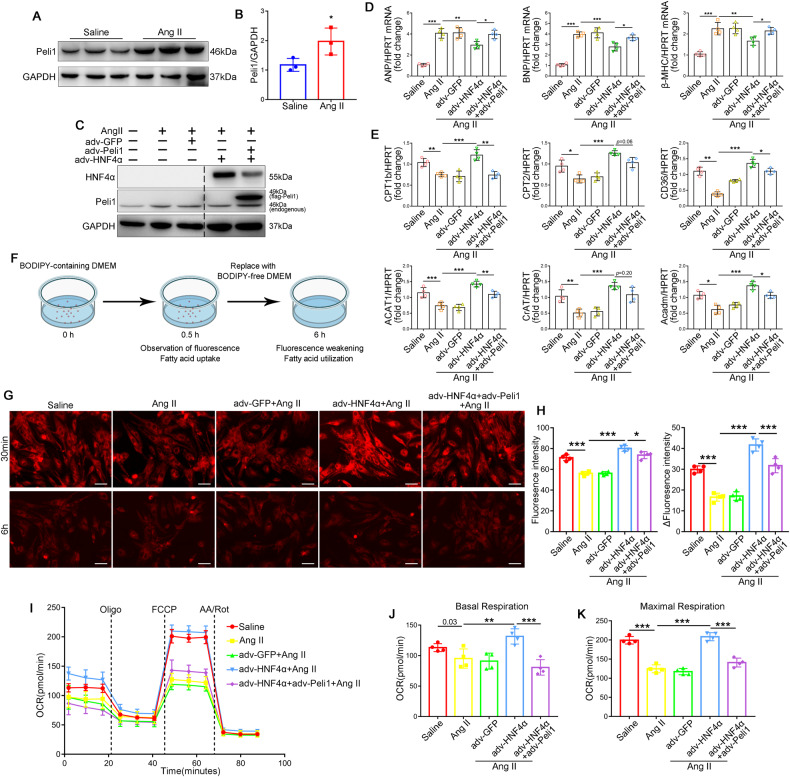
Cardiac-specific HNF4α overexpression represses Ang II-induced cardiac hypertrophy and fatty acid metabolism disorder. NRCMs were isolated from 1 to 3-day-old Sprague-Dawley rats and treated with angiotensin II (Ang II, 1 μM) for 24 hours. **A**, **B** Western blot analysis of protein levels of Peli1, GAPDH was the loading control (*n* = 3). **C**–**K** NRCMs transduced with adv-HNF4α, adv-Peli1 or adv-GFP for 24 h before the cells were treated with Ang II. **D** qRT-PCR was performed to analyze the mRNA levels of ANP, BNP and β-MHC (*n* = 4). **E** qRT-PCR was performed to analyze the mRNA levels of CPT1b, CPT2, CD36, CrAT, ACAT1 and Acadm (*n* = 4). **F** Schematic illustration of fatty acid analogue incorporation experiments. **G** Representative fluorescence graphs of fatty acid BODIPY staining in cardiomyocytes for 30 min and 6 h with BODIPY staining removed (*n* = 4). Scale bar: 200 μm. **H** Fluorescence intensity of BODIPY at 30 min (left) and the change in fluorescence intensity of BODIPY at 6 h (ΔF) compared with that at 30 min (right) (*n* = 4). **I** Oxygen consumption rate (OCR) of NRCMs with or without Ang II treatment (*n* = 4). **J**, **K** Quantification of basal respiration (left) and maximal respiration (right) of NRCMs treated with or without Ang II (*n* = 4). Values are mean ± SD. *p < 0.05, **p < 0.01, ***p < 0.001 (one-way ANOVA followed by Tukey’s test).

Based on the results of in vivo experiments, we hypothesized that Peli1 in cardiomyocytes could similarly regulate FAO in cardiomyocytes through HNF4α. To substantiate this hypothesis, we isolated NRCMs and stimulated NRCMs with Ang II to induce cardiomyocyte hypertrophy. Ang II treatment significantly upregulated Peli1 expression in NRCMs (Fig. 5A, B). Subsequently, NRCMs were infected with adv-Peli1 and adv-HNF4α. Interestingly, Peli1 overexpression led to a significant reduction in the protein expression of HNF4α (Fig. 5C), indicating that Peli1 promotes HNF4α degradation. To assess the effect of HNF4α on cardiomyocyte hypertrophy, qRT-PCR was performed to measure the expression of hypertrophy markers (ANP, BNP, and β-MHC). Overexpression of HNF4α in cardiomyocytes significantly inhibited Ang II-induced cardiomyocyte hypertrophy, while Peli1 overexpression counteracted this effect (Fig. 5D). Moreover, HNF4α overexpression increased the expression of FAO genes in NRCMs, whereas co-treatment with Peli1 overexpression adenovirus (adv-Peli1) attenuated this effect (Fig. 5E).

BODIPY is a fluorescently labeled fatty acid that mimics the transport and metabolism of native lipids in cells [27]. BODIPY has been utilized in previous studies to visualize the uptake and utilization of fatty acid [14]. The fluorescence intensity at 30 min reflected the uptake of fatty acid by cardiomyocytes, NRCMs were then washed and replaced with fresh medium without BODIPY, and the fluorescence intensity at 6 h was compared with that at the previous time to reflect the fatty acid utilization, respectively (Fig. 5F). At 30 min, compared with the saline-treated group, fatty acid uptake by NRCMs was reduced in the AngII-treated group (Fig. 5G, H). Moreover, with AngII administration, the fluorescence intensity was significantly higher in the adv-HNF4α group, but it was comparatively lower in the group treated with both adv-HNF4α and adv-Peli1 (Fig. 5G, H). Consistently, treatment with AngII reduced the change of fluorescence intensity (ΔF), which implied the utilization of fatty acid by NRCMs (Fig. 5H). Notably, ΔF at 6 h was significantly higher in the AngII combined with adv-HNF4α treatment group, while it was reduced in the group treated with both adv-HNF4α and adv-Peli1 (Fig. 5H). These results indicate that HNF4α overexpression promotes the uptake and utilization of fatty acid by NRCMs, while Peli1 overexpression inhibits this effect.

Furthermore, a palmitate oxidation stress test was performed to assess the FAO capacity of NRCMs, which was implied by the oxygen consumption rate (OCR). Compared with the Saline group, Ang II treatment diminished OCR at baseline and maximum mitochondrial respiration (Fig. 5I–K). Under Ang II stimulation, overexpression of HNF4α significantly elevated OCR in the adv-GFP group, and this effect was most reduced after the addition of oligomycin, suggesting that NRCMs with high HNF4α expression displayed higher FAO rates at baseline (Fig. 5I–K). Following the addition of FCCP, the OCR of the adv-HNF4α group was significantly higher than the other groups, indicating an enhanced maximum mitochondrial respiration during this period, reflecting a stronger FAO capacity in NRCMs of the adv-HNF4α group (Fig. 5I–K). However, both basal and maximal respiration showed a significant decrease after overexpression of Peli1 (Fig. 5I–K). These results demonstrated that overexpression of HNF4α enhanced the ability of NRCMs to utilize fatty acids for oxidative phosphorylation, while overexpression of Peli1 inhibited the effect of HNF4α.

### Peli1 promotes the ubiquitination modification of HNF4α

**Fig. 6:**
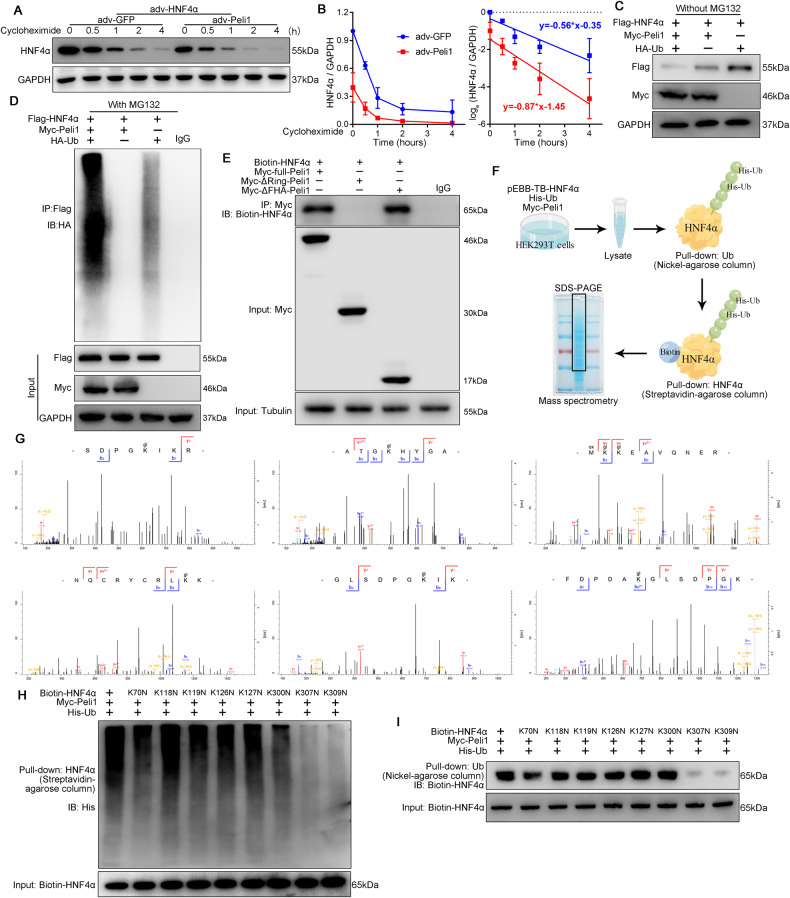
Peli1 promotes the ubiquitinated degradation of HNF4α. **A**, **B** NRCMs transduced with adv-HNF4α, adv-Peli1 or adv-GFP for 48 h, followed by treatment with 100 μM cycloheximide for 0.5, 1, 2 and 4 h. Western blot analysis of protein levels of HNF4α, GAPDH as the loading control (*n* = 3). **C**, **D** Flag-HNF4α, Myc-Peli1 and HA-Ub plasmids were transfected in HEK293T cell for 48 h. **C** Western blot analysis of protein levels of Flag and Myc, GAPDH as the loading control (*n* = 3). **D** 100 μM MG132 was added 4 h prior to protein lysate collection. Whole cell lysates were immunoprecipitated with Flag antibody, and then immunoblotted with antibodies against HA (*n* = 3). **E** Biotin-HNF4α was cotransfected with Myc-Peli1, Myc-ΔFHA-Peli1 and Myc-ΔRING-Peli1 to HEK293T cells respectively for 48 h. Whole cell lysates were immunoprecipitated with Myc antibody, and then chemiluminescence with Streptavidin-Biotin complex containing HRP, Tubulin as the loading control (*n* = 3). **F** Diagrammatic illustration for the bimolecular affinity purification for isolation of ubiquitinated HNF4α. **G** Mass spectrometric identification of HNF4α ubiquitination-modified lysine sites (K70, K118, K119, K126, K127, K300, K307 and K309). **H**, **I** Construction of HNF4α lysine locus mutant plasmids, the eight mutant plasmids were respectively cotransfected into HEK293T cells with myc-Peli1 and His-Ub plasmids for 48 h. **H** Pull-down of Biotin-HNF4α with streptavidin-agarose column, western blot analysis of protein levels of His-Ub (*n* = 3). **I** Pull-down of His-Ub with Nickel-agarose column, western blot analysis of protein levels of Biotin-HNF4α (*n* = 3).

Based on the results shown in Fig. 5C, we further investigated the mechanism by which Peli1 affects HNF4α protein expression. NRCMs infected with adv-Peli1 and adv-HNF4α were treated with cycloheximide to limit protein synthesis, and we observed a time-dependent reduction in HNF4α levels upon Peli1 overexpression (Fig. 6A, B). Similarly, when we transfected HEK293T cells with Flag-HNF4α, Myc-Peli1, and HA-Ub, we found that Peli1 reduces HNF4α protein levels (Fig. 6C). To explore whether Peli1 promotes the ubiquitination of HNF4α, we used the proteasome system inhibitor MG132 and observed that Peli1 indeed enhances the ubiquitination of HNF4α (Fig. 6D). Notably, Peli1 was unable to reduce HNF4α levels after treatment with MG132 (Fig. 6D). These results suggest that Peli1 promotes the degradation of the HNF4α via ubiquitin-proteasome system.

To identify the specific domain of Peli1 that interacts with HNF4α, we constructed plasmids encoding Myc-tagged full-length Peli1 (Myc-Full-Peli1), Peli1 with a deletion of the FHA domain (aa 276-418, Myc-ΔFHA-Peli1), and Peli1 with a deletion of the RING-like domain (aa 1-289, Myc-ΔRING-Peli1) (Supplementary Fig. 1A–C). These three plasmids were individually cotransfected with Biotin-HNF4α plasmid into HEK293T cells, and co-immunoprecipitation (Co-IP) experiments revealed an interaction between Full-Peli1 or ΔFHA-Peli1 and HNF4α, while no interaction was observed between HNF4α and ΔRING-Peli1 (Fig. 6E). These findings demonstrate that Peli1 binds to HNF4α through its RING-like domain.

We further explored the lysine sites of ubiquitination modification of HNF4a. His-tagged Ub plasmid (His-Ub), an HNF4α plasmid containing TEV cleavage site and biotinylated peptide (pEBB-TB-HNF4α) were designed, and both plasmids were cotransfected with Myc-Peli1 plasmid in HEK293T cells. The His-Ub and biotinylated HNF4α were purified by bimolecular affinity purification assay, and a large amount of purified HNF4α protein modified by ubiquitination was obtained and analyzed by liquid chromatography-mass spectrometry LC-MS (Fig. 6F). Eight lysine sites were identified, which were lysine 70 site (K70), K118, K119, K126, K127, K300, K307 and K309 (Fig. 6G). Construct 8 plasmids with lysine (K) mutated to aspartic acid (N), respectively. The mutant plasmids were cotransfected with Myc-Peli1 and His-Ub plasmids in HEK293T cells, showing that ubiquitination modification of HNF4α K307N and K309N mutants were significantly inhibited (Fig. 6H). Similarly, mutations at the K307 and K309 sites resulted in a significant decrease in HNF4α binding to His-Ub (Fig. 6I). These results suggest that Peli1 promotes the ubiquitination modification of K307 and K309 residues of HNF4α.

We screened from the U.S. FDA Drugs Database using the Peli1 RING-like domain as a pocket, and selected Bezafibrate as the Peli1 E3 ubiquitin ligase activity inhibitor. To investigate the effect of inhibition of Peli1 activity on HNF4a, HEK293T cells were transfected with Flag-HNF4α, Myc-Peli1 and His-Ub plasmids, and examined for Flag-tag protein. The results showed that Peli1 resulted in a decrease in Flag expression, but Bezafibrate treatment inhibited this change (Supplementary Fig. 2A). Meanwhile, Peli1-mediated ubiquitination of HNF4α was significantly inhibited by the administration of Bezafibrate (Supplementary Fig. 2B). These results suggest that inhibition of Peli1 E3 ubiquitin ligase activity using Bezafibrate inhibits the role of Peli1 in promoting the degradation of the HNF4α via ubiquitin-proteasome system.

## Discussion

The present study has revealed a previously unknown role of Peli1 in the ubiquitination of HNF4α, which contributes to impaired FAO in the hypertrophied heart. We observed that deletion of Peli1 significantly increased HNF4α expression and promoted the transcription of FAO-related target genes regulated by HNF4α. This, in turn, enhanced cardiomyocyte FAO, attenuated pressure overload-induced pathological myocardial hypertrophy, and improved cardiac function. Our findings suggest that Peli1 conjugates ubiquitin chains to HNF4α at residues K307 and K309, leading to the degradation of HNF4α through the ubiquitin-proteasome system. Thus, Peli1 plays a crucial role in TAC-induced pathological myocardial hypertrophy by promoting the ubiquitinated degradation of HNF4α and affecting FAO (Graphical Abstract).

Metabolic shifts play a crucial role in the underlying mechanisms of cardiac diseases, including pathological myocardial hypertrophy [5]. One significant alteration is the reduction in FAO, while the myocardium tends to rely more on glucose for energy supply, leading to decreased oxidative metabolism and a decline in total ATP production (approximately 30% decrease) [10, 28]. Among various fuel substrates, the oxidation of long-chain fatty acids (LCFAs) generates the highest number of ATP molecules. As the organ with the highest energy consumption per gram of tissue, the heart primarily relies on LCFAs as its main fuel source [5]. Consequently, the energy supply of the heart is heavily dependent on FAO, and any disruption in this process can significantly impact myocardial energy production and contribute to the development of myocardial injury. In pathological cardiac hypertrophy, the impairment of cardiac function is often accompanied by a decrease in FAO rate [29–31]. However, early studies tended to inhibit fatty acid utilization by the myocardium and enhance glucose utilization to prevent and treat pathological myocardial hypertrophy, as oxidation of equivalent fatty acids consumes more oxygen than glucose. For instance, a study by H. Rupp et al. in 1997 showed that etomoxir, an inhibitor of CPT-1, significantly improved pressure overload-induced heart failure caused by increased pressure on the heart [32]. However, recent studies have challenged this perspective. Yang, Q et al. found that CPT1b deficiency actually exacerbated pressure overload-induced heart hypertrophy [33]. Similarly, as a regulator of cardiomyocyte FAO, knockout of PPARα worsened pressure overload-induced heart failure [7], while PPARα agonists improved pathological myocardial hypertrophy [34]. CD36, which plays a role in the uptake of LCFAs in the myocardium, has been shown to inhibit FAO in cardiomyocytes when knocked out, but this also significantly worsened pathological myocardial hypertrophy [35]. Conversely, knockout of acetyl coenzyme A carboxylase 2 (ACC2) enhanced fatty acid utilization, decreased glucose utilization, and improved pathological cardiac hypertrophy due to pressure overload, resulting in increased phosphocreatine content in cells [9]. These findings collectively suggest that maintaining normal mitochondrial FAO in cardiomyocytes during the development of pathological cardiac hypertrophy may confer a protective effect. Our study aligns with these findings and confirms that increased expression of HNF4α promotes the transcription of FAO genes in pathological myocardial hypertrophy, leading to elevated FAO in cardiomyocytes and an improvement in myocardial hypertrophy.

HNF4α belongs to the nuclear receptor superfamily of ligand-dependent transcription factors. Classical promoter analysis has identified functional HNF4α binding sites in over 140 genes involved in glucose, lipid, and amino acid metabolism [36]. HNF4α plays a crucial role in maintaining metabolic homeostasis and regulating the transcription of genes involved in hepatic lipid metabolism. Deletion of HNF4α in the liver leads to hepatic lipid accumulation, severe steatosis, and even the development of fatty liver [12, 37]. HNF4α also regulates the transcription of CPT1, the rate-limiting enzyme for long-chain fatty acid oxidation, as well as genes involved in acylcarnitine metabolism, including Octn2, Cact, CPT2, and CrAT [11, 13, 38–40]. In Drosophila, HNF4α acts as a sensor for free fatty acids and is essential for lipid catabolism and beta-oxidation for energy production [41]. In our study, we observed a modest increase in HNF4α after TAC, which did not lead to a significant elevation in the transcript level of FAO-related target genes. We speculate that this slight increase may represent a compensatory change. However, deletion of Peli1 resulted in a significant increase in HNF4α protein levels and downstream target genes expression. This suggests that Peli1, acting as an E3 ubiquitin ligase, promotes the degradation of HNF4α through the ubiquitin-proteasome system, inhibiting the compensatory effect of HNF4α. Furthermore, we conducted experiments with myocardial-specific overexpression of HNF4α in mice using rAAV9-HNF4α, which demonstrated attenuated myocardial hypertrophy and improved cardiac function following TAC. These findings provide further evidence that enhanced FAO mediated by HNF4α can ameliorate pressure overload-induced cardiac hypertrophy.

Post-translational modifications can impact the protein levels of HNF4α. In various conditions such as high-fat diet (HFD)-fed mice, db/db mice, and palmitate-treated hepatoma cells, acetylation modification of HNF4α increases, leading to its protein degradation. However, Sirt2, a deacetylase, interacts with HNF4α, inhibits acetylation modification specifically at the K458 residue, and enhances the protein’s stability. This process effectively suppresses hepatic lipid accumulation, steatosis, and metabolic disturbances [15]. Additionally, SUMO2 facilitates SUMO-based modifications of HNF4α, promoting its subsequent degradation through the proteasome pathway. Consequently, HNF4α protein level decrease due to this modification [42]. In our study, we focused on the ubiquitination modification of HNF4α and demonstrated that Peli1 promotes HNF4α degradation via the proteasome, thereby negatively regulating its protein level. Moreover, we identified specific ubiquitination modification sites at residues K307 and K309. Bezafibrate blocks HNF4α ubiquitin-proteasome pathway degradation by inhibiting Peli1 activity as an E3 ubiquitin ligase. This not only enhances the pharmacological effects of bezafibrate and offers a foundation for clinical medication guidance, but it also adds an investigation instrument for the E3 ubiquitin ligase Peli1.

Peli1, an E3 ubiquitin ligase, has been primarily associated with TLR signaling and various intracellular inflammatory and innate immune signaling pathways [43–46]. However, the non-inflammatory regulatory role of Peli1 in cell signaling remains incompletely characterized. Consistent with previous studies, our research observed the significant impact of Peli1 on the transcriptome of signaling processes in cardiac hypertrophy. However, our results also revealed an unexpected pleiotropic effect of Peli1 on metabolic processes, indicating its broader functional implications. This finding aligns with a recent study demonstrating that Peli1 negatively regulates the metabolic reprogramming of CD8 + T cells, where Peli1 deficiency led to increased baseline OCR and stress OCR [20]. By investigating the role and mechanism of Peli1 in regulating lipid metabolism, we aimed to comprehensively elucidate the molecular function of Peli1. The RNA-seq results further suggested that the extensive and potent functions of Peli1 have yet to be fully explored.

Nevertheless, our study has several limitations. The majority of experiments were conducted using mouse models, and while we extensively validated the regulation of FAO by HNF4α at the cellular level, monitoring changes in myocardial FAO in vivo remains challenging. Furthermore, the impact on FAO in in vivo experiments was only demonstrated by the mRNA of genes related to fatty acid metabolism due to few detection methods.

In conclusion, the present study provides compelling evidence that the regulatory effect of Peli1 on HNF4α plays a crucial role in the impairment of FAO during pathological myocardial hypertrophy caused by pressure overload. This novel finding offers valuable insights and a theoretical foundation for the development of intervention strategies and clinical treatments targeting pathological cardiac hypertrophy. These findings pave the way for potential advancements in the field and hold promise for improving patient outcomes in the future.

### Supplementary information

Supplementary material

Reproducibility checklist

Supplemental Material for original western blots

## Data Availability

The raw sequence data reported in this paper have been deposited in the Genome Sequence Archive (Genomics, Proteomics & Bioinformatics 2021) in National Genomics Data Center (Nucleic Acids Res 2022), China National Center for Bioinformation / Beijing Institute of Genomics, Chinese Academy of Sciences (GSA: CRA014070) that are publicly accessible at https://ngdc.cncb.ac.cn/gsa.
