# Supplementary material for: HNF4α ubiquitination mediated by Peli1 impairs FAO and accelerates pressure overload-induced myocardial hypertrophy
Source: Cell Death Dis. 2024 Feb 12;15(2):135. doi: 10.1038/s41419-024-06470-7 (PMC10861518; doi:10.1038/s41419-024-06470-7)
Supplement: Supplementary file 1 — Supplementary material [file 41419_2024_6470_MOESM1_ESM.pdf]

HNF4α ubiquitination mediated by Peli1 impairs FAO and accelerates pressure overload-induced myocardial hypertrophy

Yuxing Hou [#, 1], Pengxi Shi [#, 1], Haiyang Du [1], Chenghao Zhu [1], Chao Tang [1, 2], Linli Que [1], Guoqing Zhu [3], Li Liu [4], Qi Chen [1], Chuanfu Li [5], Guoqiang Shao [6, *], Yuehua Li [1, *], Jiantao Li [1, *]

[1] Key Laboratory of Targeted Intervention of Cardiovascular Disease, Collaborative Innovation Center for Cardiovascular Disease Translational Medicine, School of Basic Medical Science, Nanjing Medical University, Nanjing 211166, China.

[2] Department of Pathology and Pathophysiology, School of Medicine & Holistic Integrative Medicine, Nanjing University of Chinese Medicine, Nanjing 210023, China.

[3] Key Laboratory of Targeted Intervention of Cardiovascular Disease, Department of Physiology, Nanjing Medical University, Nanjing 211166, China.

[4] Department of Geriatrics, the First Affiliated Hospital of Nanjing Medical University, Nanjing 210029, China.

[5] Department of Surgery, East Tennessee State University, Campus Box 70575, Johnson City, TN 37614-0575, USA.

[6]Department of nuclear medicine, Nanjing First Hospital, Nanjing Medical University, Nanjing 210029, China.

[#] These authors contributed equally to this work.

***Correspondence:** Jiantao Li, Key Laboratory of Targeted Intervention of Cardiovascular Disease, Collaborative Innovation Center for Cardiovascular Disease Translational Medicine, School of Basic Medical Science, Nanjing Medical University, Nanjing, Jiangsu Province, 211166, China. Tel: 86-025-86869331. **E-mail:** ljt@njmu.edu.cn

Correspondence: Yuehua Li, Key Laboratory of Targeted Intervention of Cardiovascular Disease, Collaborative Innovation Center for Cardiovascular Disease Translational Medicine, School of Basic Medical Science, Nanjing Medical University, Nanjing, Jiangsu Province, 211166, China. Tel: 86-025-86869331. **E-mail:** yhli@njmu.edu.cn

Correspondence: Guoqiang Shao, Department of nuclear medicine, Nanjing First Hospital, Nanjing Medical University, Nanjing, Jiangsu Province, 210029, China. Tel: 86-025-52271000. **E-mail:** guoqiangshao@163.com

Running title: Peli1 regulates FAO through ubiquitination of HNF4α

Supplementary Figures

Supplementary Figure 1

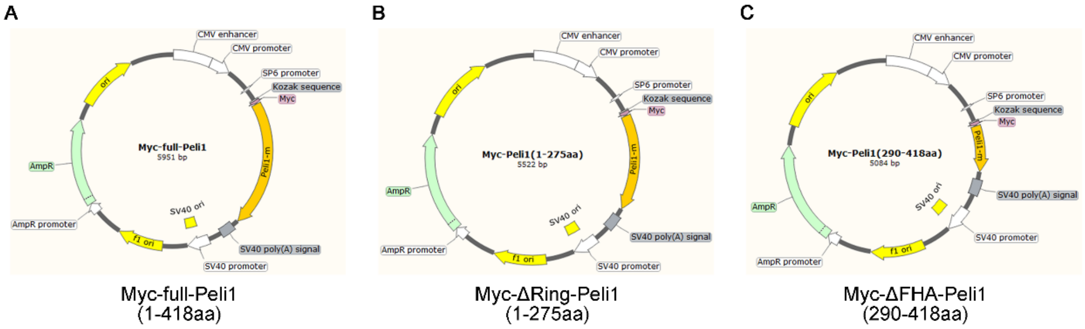

Supplemental Figure 1. Peli1 plasmid profile. **(A)** Myc-tagged full-length Peli1 (Myc-Full-Peli1) plasmid. **(B)** Peli1 with a deletion of the FHA domain (aa 276–418, Myc-ΔFHA-Peli1) plasmid. **(C)** Peli1 with a deletion of the RING-like domain (aa 1–289, Myc-ΔRING-Peli1) plasmid.

Supplementary Figure 2

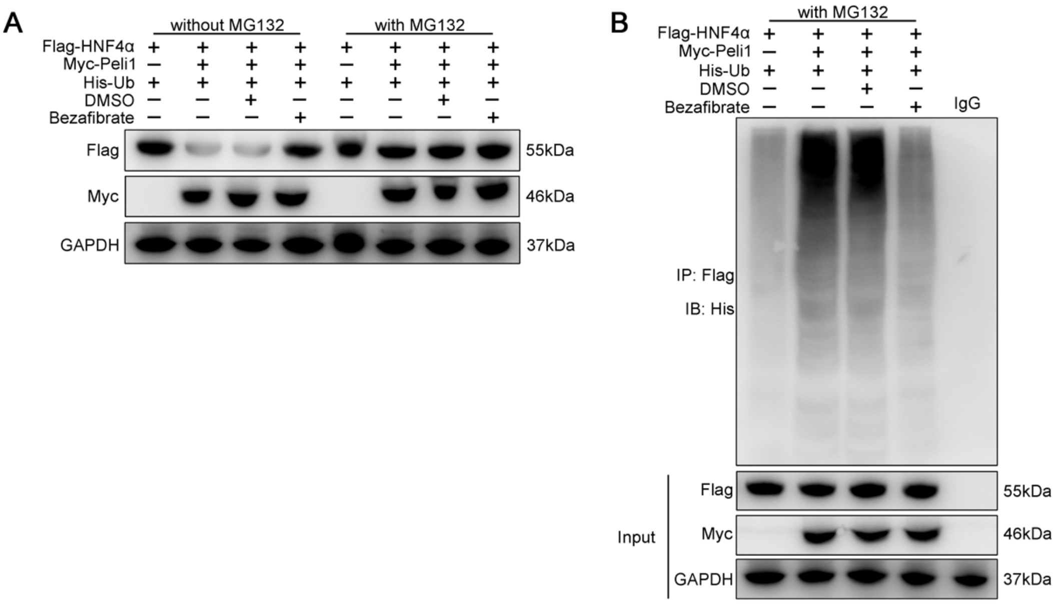

Supplemental Figure 2. HEK293T cells were transfected with Flag-HNF4α, Myc-Peli1, and HA-Ub and treated with 100μM bezafibrate with or without MG132 after 44 hours, and protein lysates were collected after 48 hours. **(A)** Western blot analysis revealed the presence of Flag and Myc. **(B)** 100μM MG132 was added 4 hours before protein extraction, and the expression of Flag and Myc, as well as the amount of HNF4α ubiquitination, were detected by Western blot. (*n=3*).

Supplementary Table 1. ***HNF4α binding sites in transcriptional regulatory regions of genes in Fig2D.***

Gene ID	Gene	Binding Sites for HNF4α			
		Site 1	Site 2	Site 3	Site 4
12895	'Cpt1b'	gTGACCTTTTccc			
12896	'Cpt2'	aTGAACTTTTtca			
12491	'Cd36'	gttCAAACTTCAc	attAAAAGTTCTt		
12908	'Crat'	TGGCCTCTGTCTc			
110446	'Acat1'	tTTACCCTTGGCCt			
11364	'Acadm'	ctgCAAAGACCAc			
12642	'Ch25h'	gTGAGCCTTTacg			
13106	'Cyp2e1'	cTGACCTATGaat	ctcCAAGGGTCAg		
13113	'Cyp3a13'	tTGAACTTTGctc	aTGTACCTTGact	aTGAACCTTTtcc	aTAACCTTTGtac
13116	'Cyp46a1'	ctgCTAAGCTCAg			
15485	'Hsd17b1'	gAGACCTTTGcct			
171281	'Acot3'	aggGAAAGGTCAt	agtTAAAGATCAg		
18618	'Pemt'	aggCAAATTTCAc			
18784	'Pla2g5'	attCACAGTTCAc			
19223	'Ptgis'	gggCACAGCTCAg	cTGGCCTTTGtct	ggaCAAGGTTTAc	aggCTAAGTCCAt
26897	'Acot1'	gTGAGCTTTGaaa			
329502	'Pla2g4e'	aggCAAGGTTAAt			
93898	'Cers1'	gTGGACCTTTccc	cTGATCTTTGgac		

Binding sites for HNF4α in the transcriptional regulatory regions of differentially expressed genes related to "lipid metabolic process" were identified by analysis of rVista2.0 database. The upper case represents the transcription factor binding sites.
