# Supplementary material for: HNF4α ubiquitination mediated by Peli1 impairs FAO and accelerates pressure overload-induced myocardial hypertrophy
Source: Cell Death Dis. 2024 Feb 12;15(2):135. doi: 10.1038/s41419-024-06470-7 (PMC10861518; doi:10.1038/s41419-024-06470-7)

Figure 1A + Figure 2E

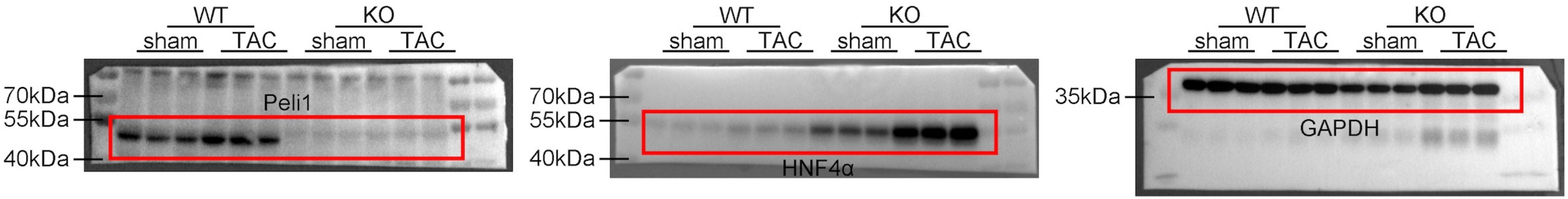

Figure 2H

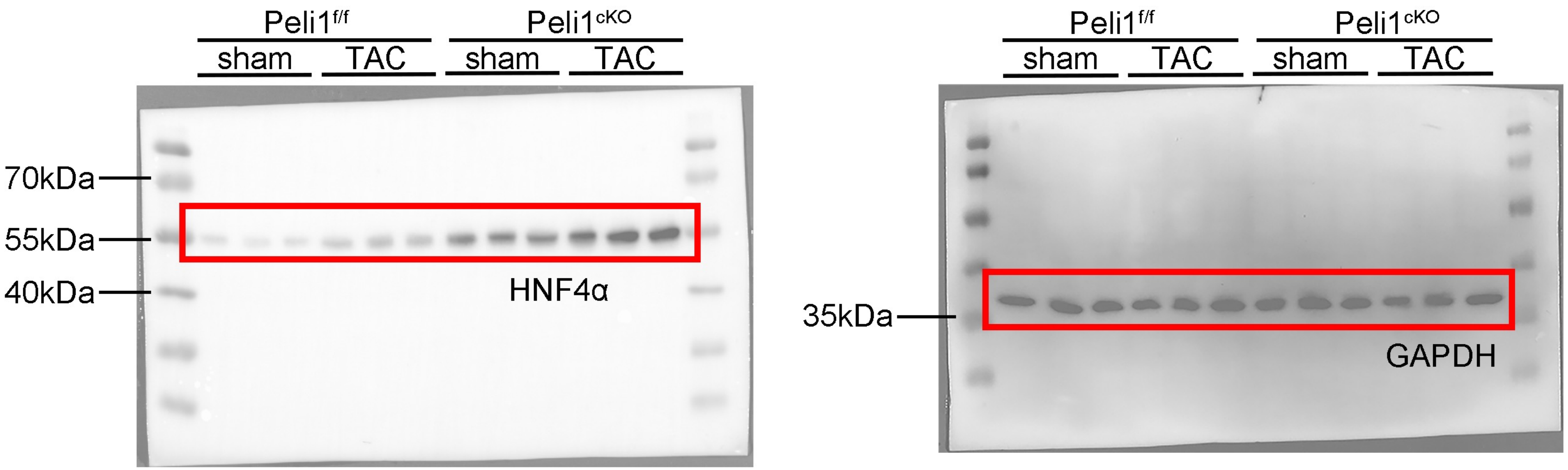

Figure 4A

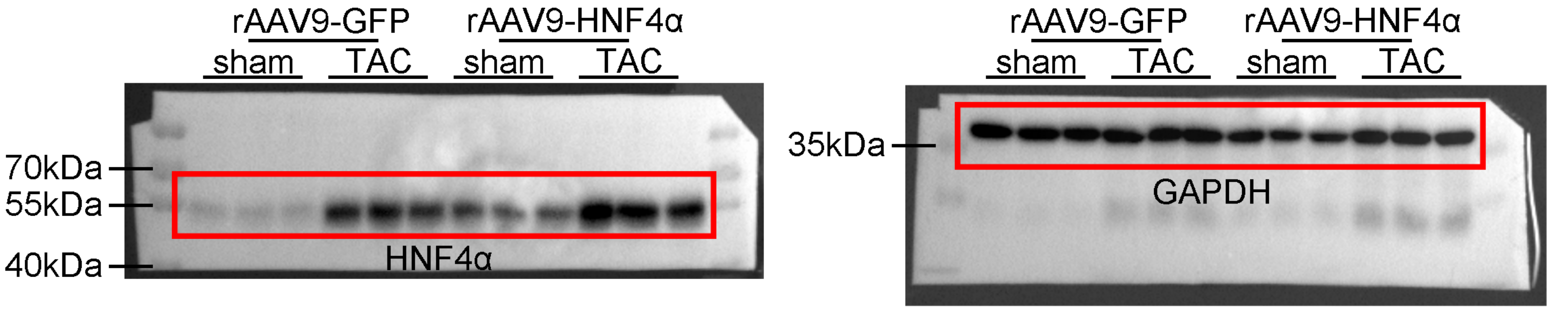
rAAV9-GFP
rAAV9-HNF4α
sham
TAC
sham
TAC
70kDa
55kDa
40kDa
HNF4α
rAAV9-GFP
rAAV9-HNF4α
sham
TAC
sham
TAC
35kDa
GAPDH

Figure 5A

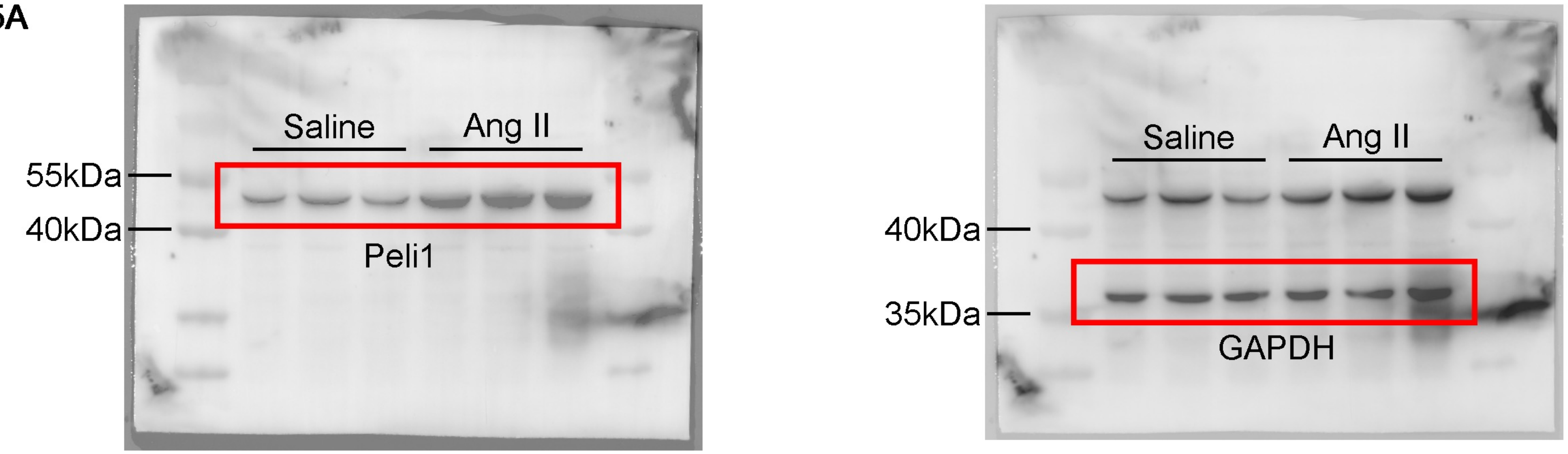

Figure 5C

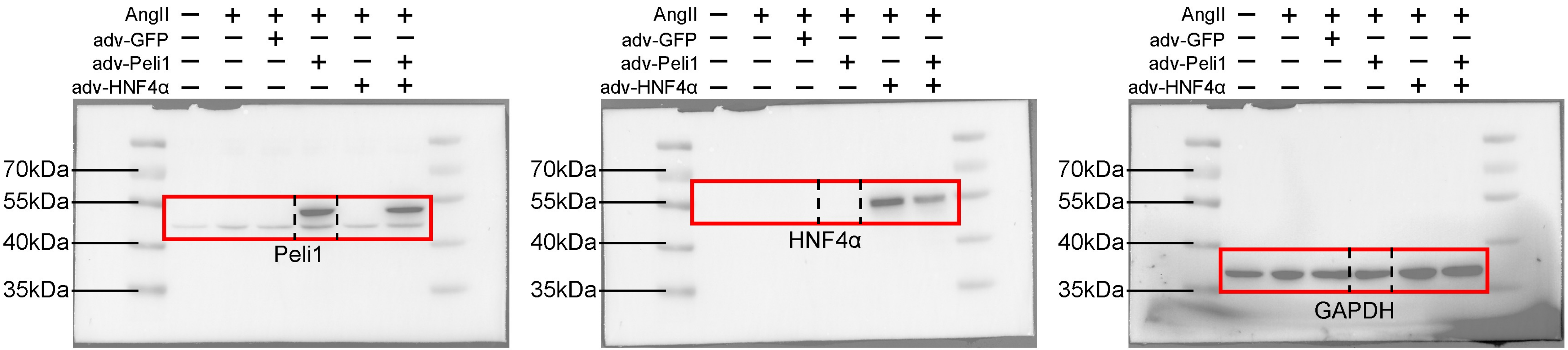

Figure 6A

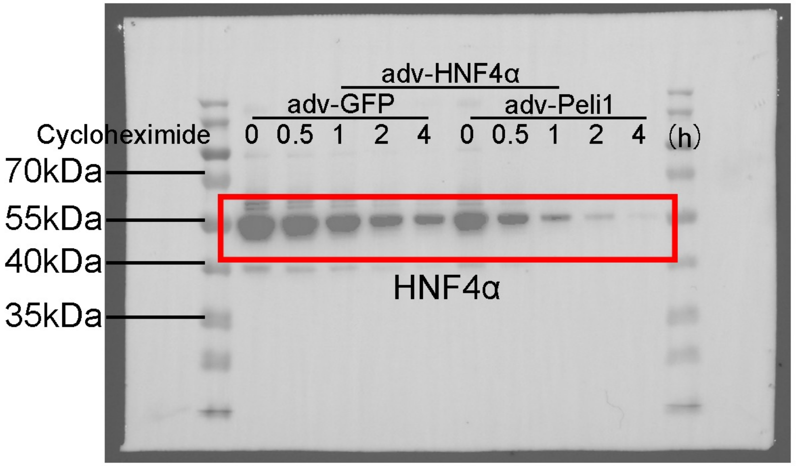

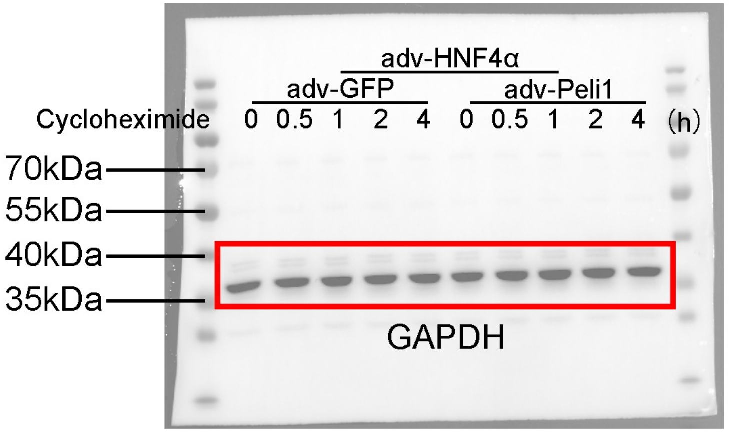

Figure 6C

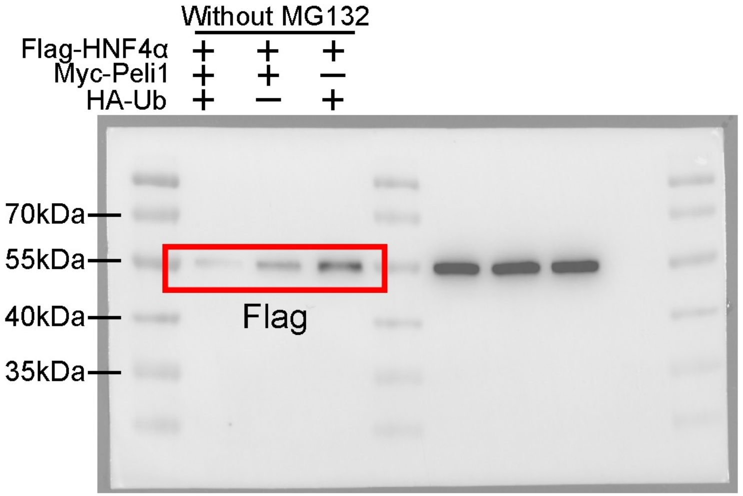

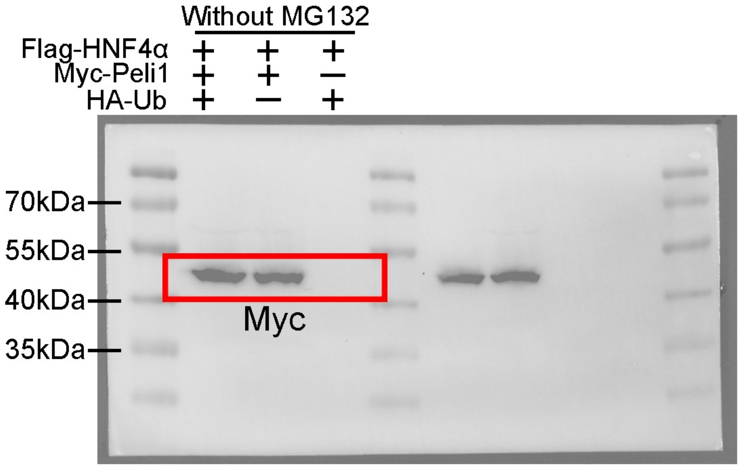

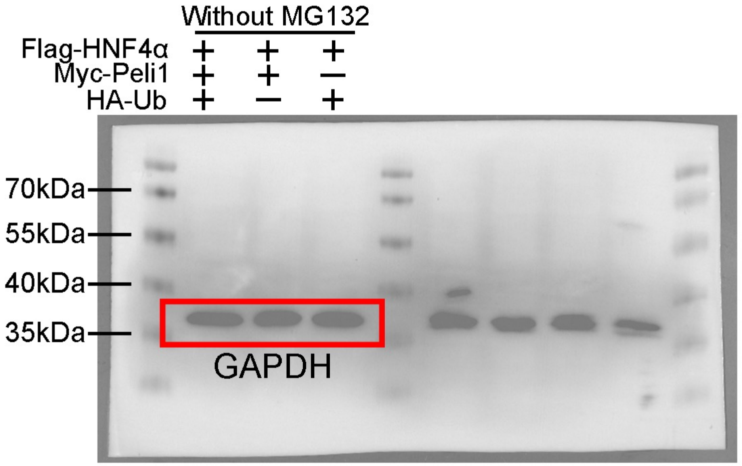

Figure 6D

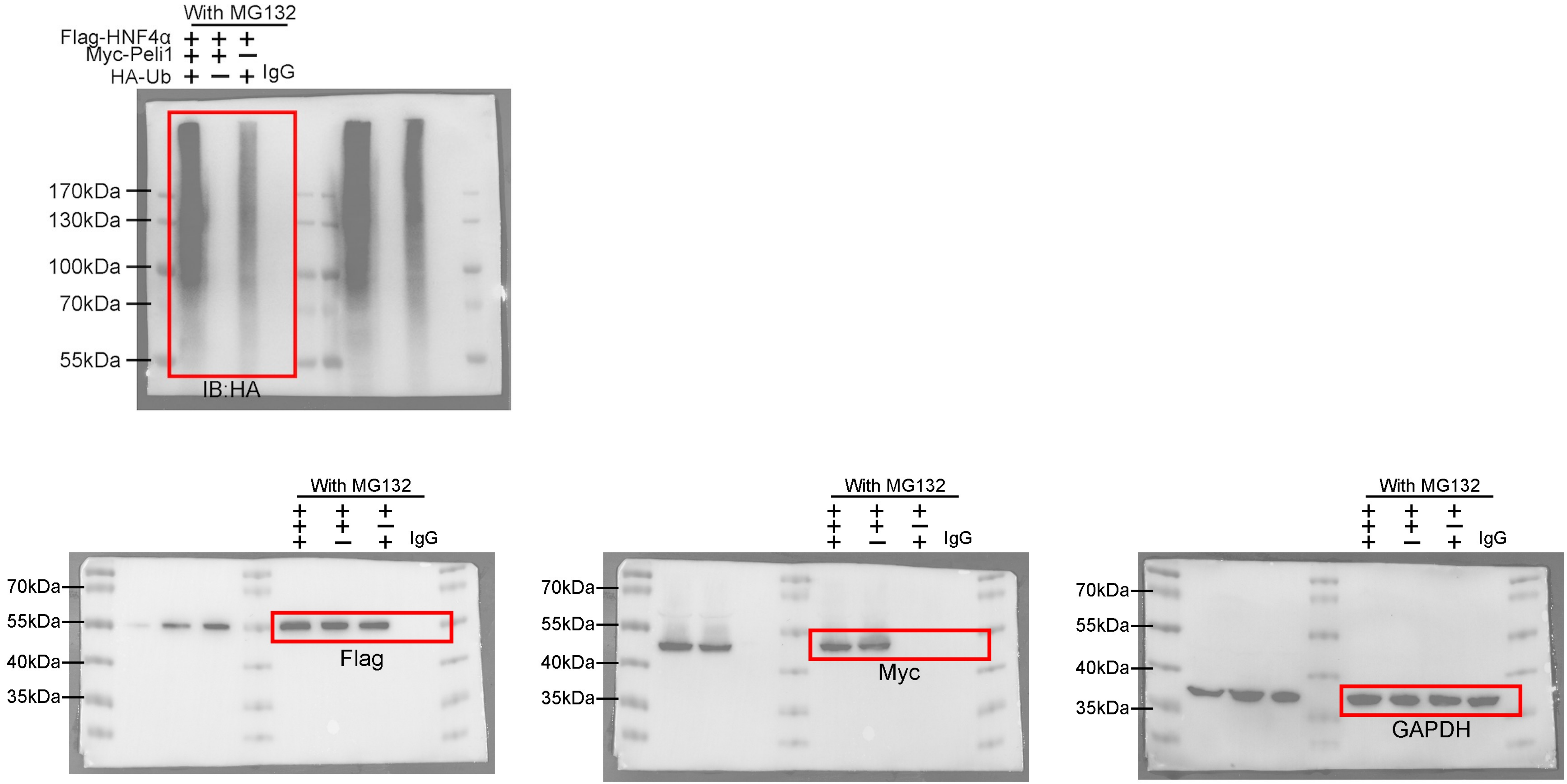
With MG132
Flag-HNF4α + + +
Myc-Peli1 + + −
HA-Ub + − + IgG
170kDa
130kDa
100kDa
70kDa
55kDa
IB:HA
70kDa
55kDa
40kDa
35kDa
Flag
Myc
GAPDH

Figure 6E

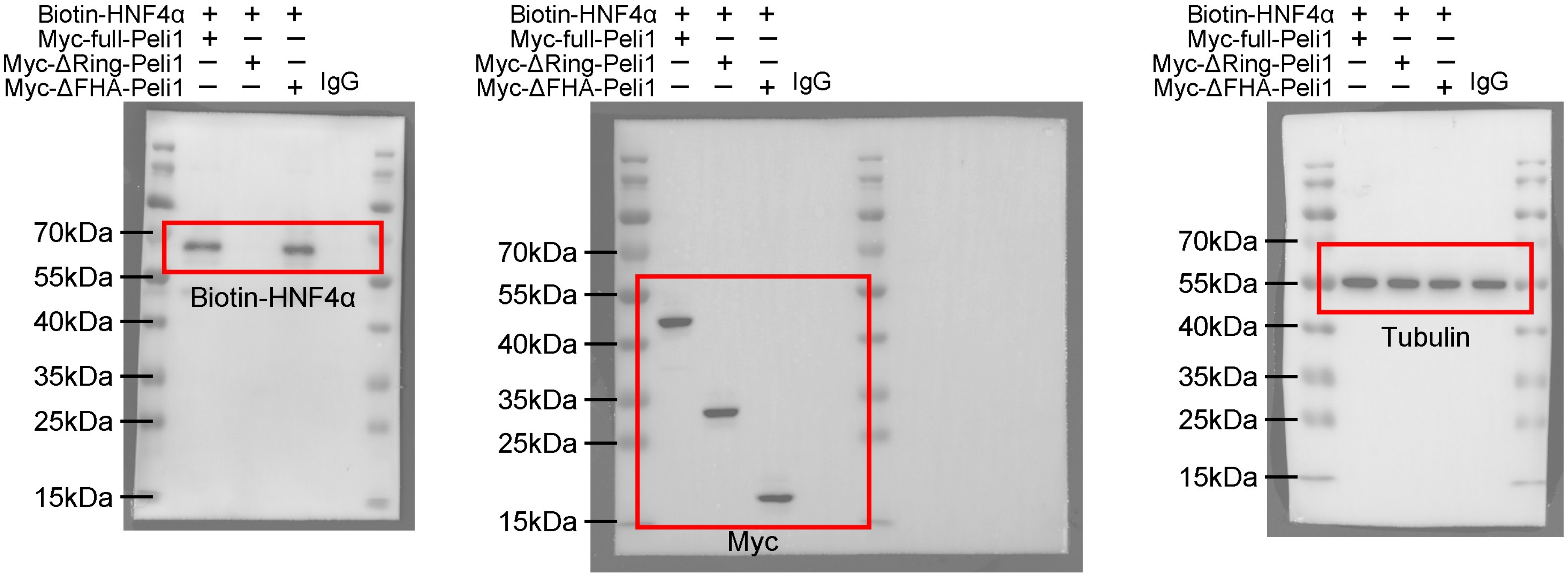
Biotin-HNF4α + + +
Myc-full-Peli1 + − −
Myc-ΔRing-Peli1 − + −
Myc-ΔFHA-Peli1 − − + IgG
70kDa
55kDa
40kDa
35kDa
25kDa
15kDa
Biotin-HNF4α
Biotin-HNF4α + + +
Myc-full-Peli1 + − −
Myc-ΔRing-Peli1 − + −
Myc-ΔFHA-Peli1 − − + IgG
70kDa
55kDa
40kDa
35kDa
25kDa
15kDa
Myc
Biotin-HNF4α + + +
Myc-full-Peli1 + − −
Myc-ΔRing-Peli1 − + −
Myc-ΔFHA-Peli1 − − + IgG
70kDa
55kDa
40kDa
35kDa
25kDa
15kDa
Tubulin

Figure 6H

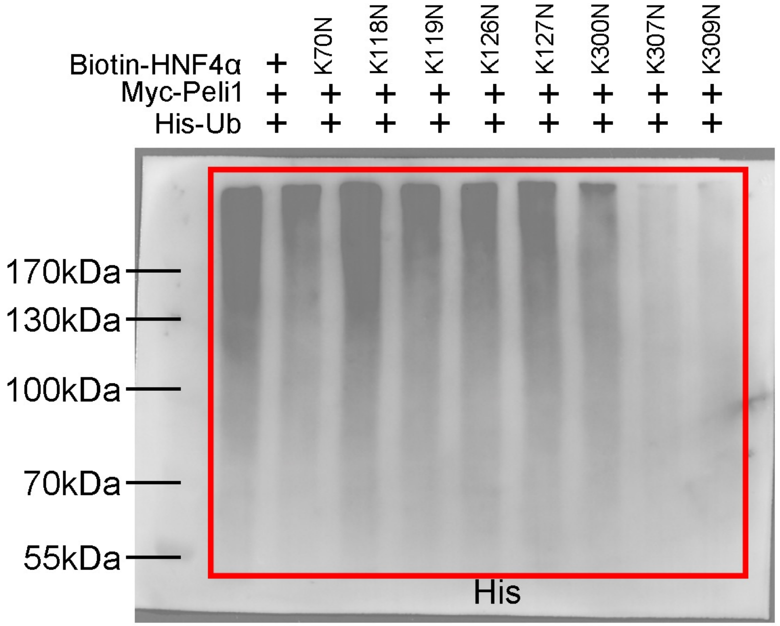

Biotin-HNF4α
Myc-Peli1
His-Ub
K70N
K118N
K119N
K126N
K127N
K300N
K307N
K309N
170kDa
130kDa
100kDa
70kDa
55kDa
His

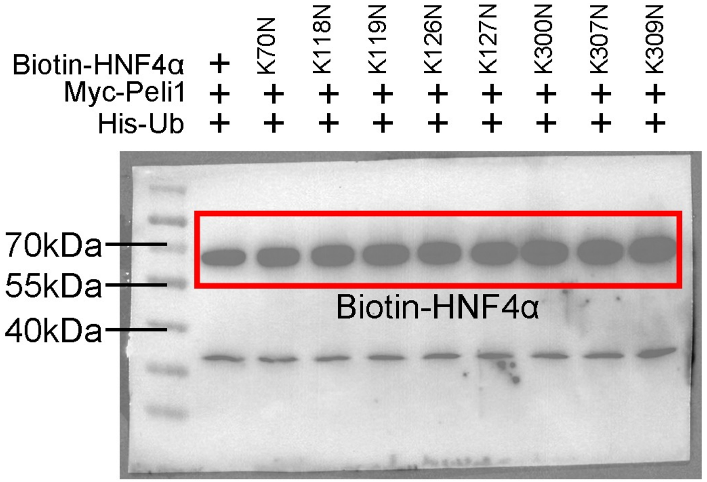

Biotin-HNF4α
Myc-Peli1
His-Ub
K70N
K118N
K119N
K126N
K127N
K300N
K307N
K309N
70kDa
55kDa
40kDa
Biotin-HNF4α

Figure 6I

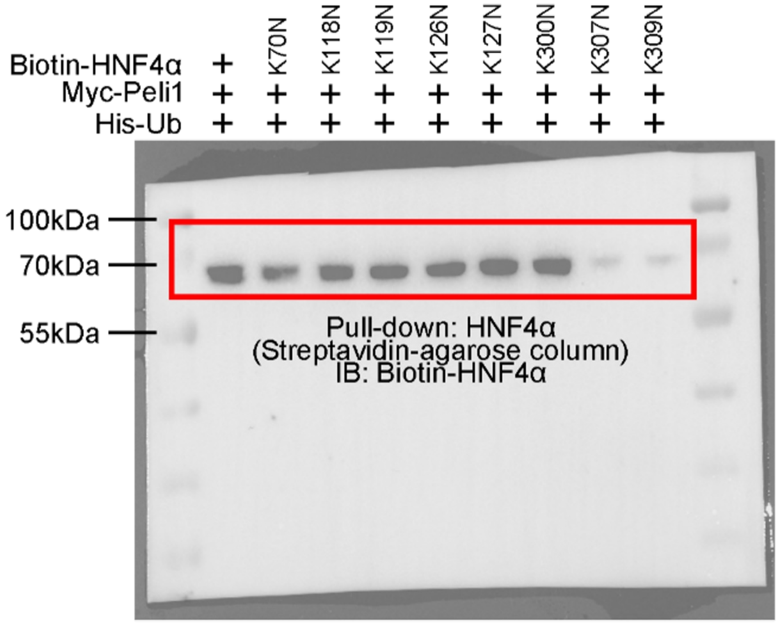
Biotin-HNF4α
Myc-Peli1
His-Ub
K70N
K118N
K119N
K126N
K127N
K300N
K307N
K309N
100kDa
70kDa
55kDa
Pull-down: HNF4α
(Streptavidin-agarose column)
IB: Biotin-HNF4α

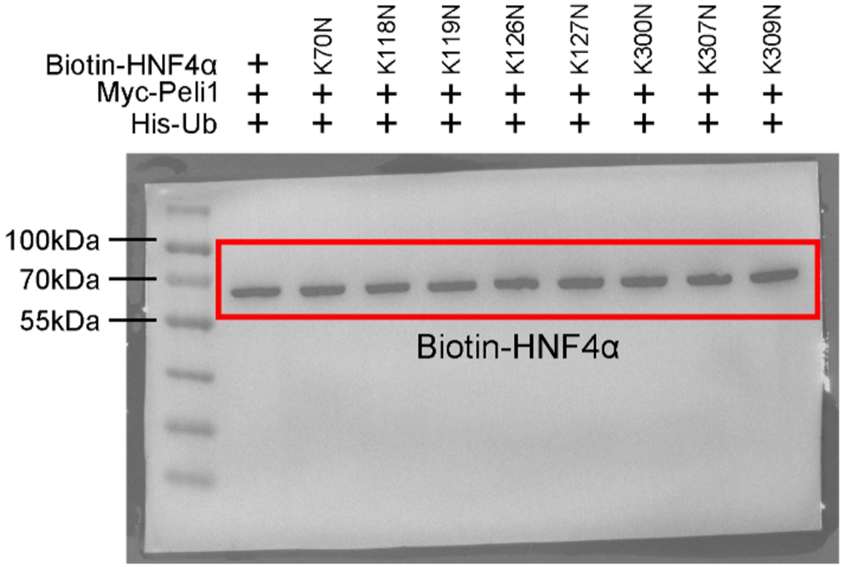
Biotin-HNF4α
Myc-Peli1
His-Ub
K70N
K118N
K119N
K126N
K127N
K300N
K307N
K309N
100kDa
70kDa
55kDa
Biotin-HNF4α

Supplementary Figure 2A

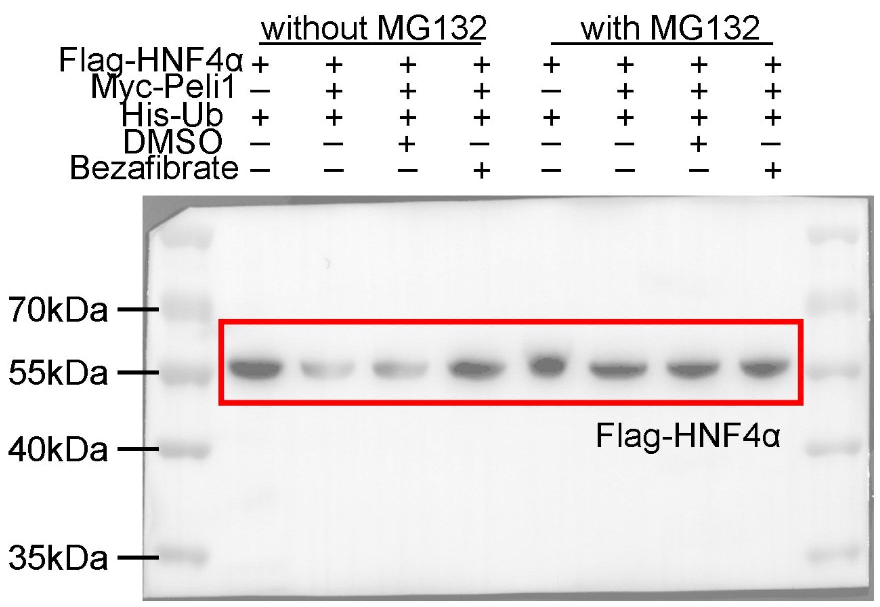

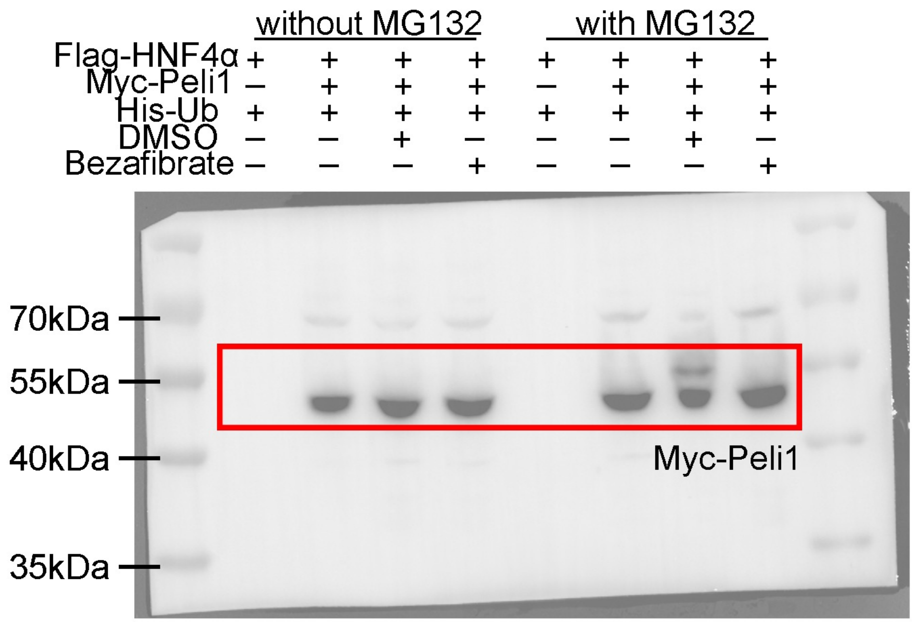

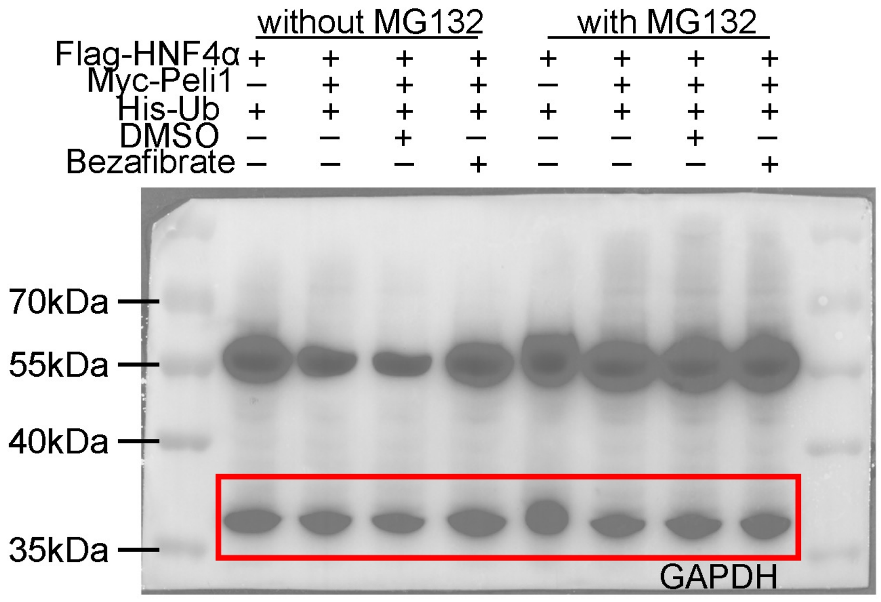

Supplementary Figure 2B

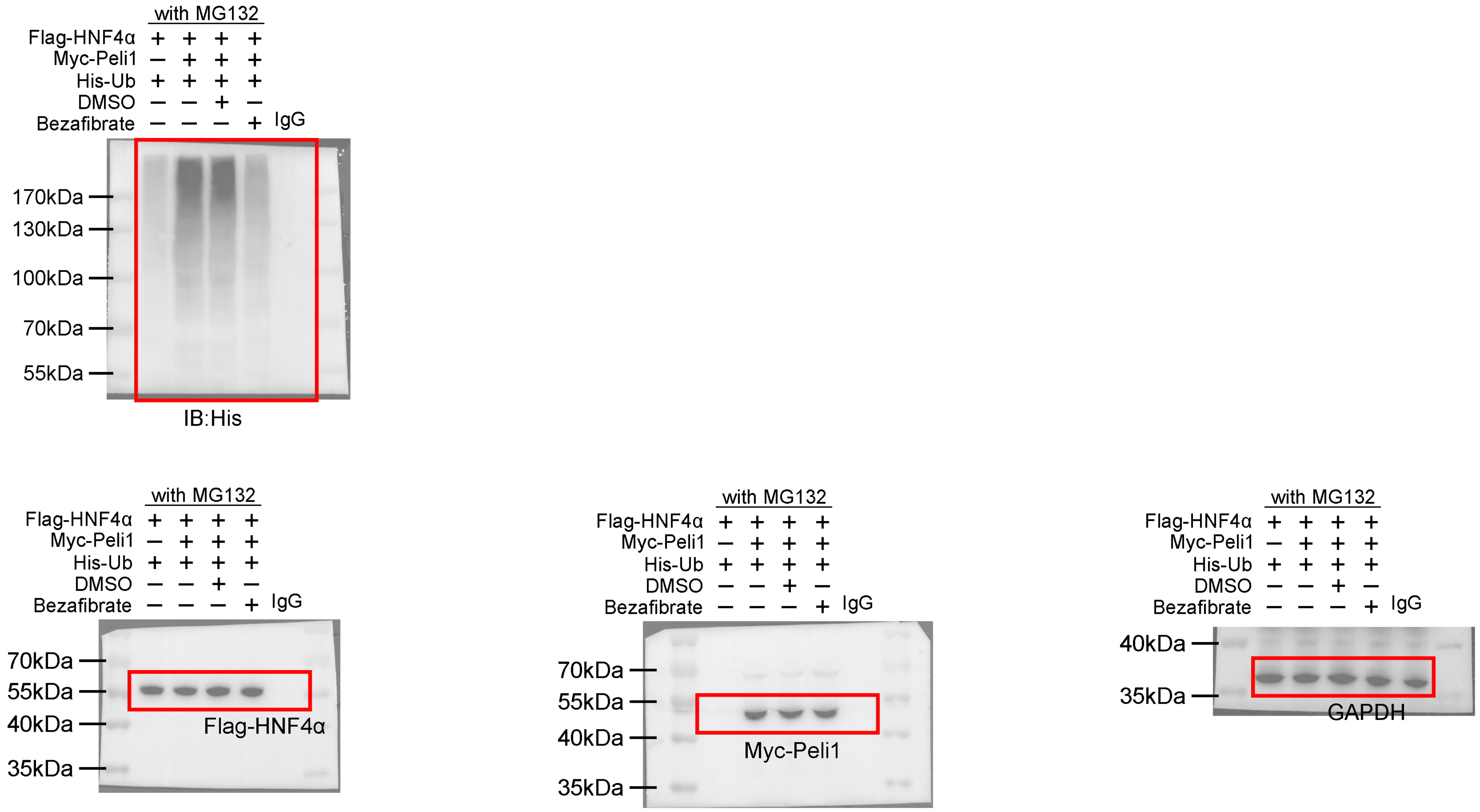

Supplement: Supplementary file 3 — Supplemental Material for original western blots [file 41419_2024_6470_MOESM3_ESM.pdf]
